# Validation of single nucleotide polymorphism markers associated with hydrogen cyanide, total carotenoid and dry matter contents in cassava (*Manihot esculenta* Crantz)

**DOI:** 10.3389/fpls.2026.1912608

**Published:** 2026-08-07

**Authors:** Bismark Anokye, Bunmi Olasanmi, Adenike Ige, Hapson Mushoriwa, Emmanuel Oladeji Alamu, Peter Amoah, Elizabeth Parkes

**Affiliations:** 1 Cassava Breeding Unit, International Institute of Tropical Agriculture (IITA), Ibadan, Nigeria; 2 Pan African University Life and Earth Sciences Institute, Ibadan, Nigeria; 3 Department of Soil and Crop Sciences, Texas Agricultural and Mechanical University (A&M) University, College Station, TX, United States; 4 Department of Crop and Horticultural Sciences, University of Ibadan, Ibadan, Nigeria; 5 Department of Crop Production and Landscape Management, University of Ebonyi State, Abakaliki, Nigeria

**Keywords:** carotenoid biofortification, dry matter content, hydrogen cyanide, KASP genotyping, marker-assisted selection, SNP markers

## Abstract

Enhancing total carotenoid content (TCC) and dry matter content (DMC) while simultaneously reducing hydrogen cyanide (HCN) are key objectives in cassava breeding. Although numerous SNP markers have been identified through genome-wide association studies (GWAS) as promising tools for accelerating improvement of quality traits, they have not been widely adopted in marker-assisted selection (MAS) due to limited validation across diverse genetic backgrounds. To address this gap, we validated nine previously reported single nucleotide polymorphism (SNP) markers associated with TCC, DMC, and HCN using 435 genotypes from two independent cassava breeding populations: a HarvestPlus Seedling Nursery (SN) population from IITA and Advanced Yield Trial (AYT) at University of Ibadan (UIC). The markers were evaluated using Kompetitive allele-specific PCR (KASP) genotyping across a subset of 360 genotypes drawn from HarvestPlus SN population, its progenitors, and selected UIC lines. The markers demonstrated high genotyping efficiency, with call rates exceeding 96% and clear allelic discrimination. Trait relationships varied across populations, with TCC and DMC showing a negative correlation in AYT (r = −0.26**) and a positive correlation in SN (r = 0.37***). Marker S1_24155522 showed a strong and significant association with TCC, while S8_25598183 exhibited a moderate effect. For DMC, marker S1_24197219 displayed limited segregation, with the unfavorable allele appearing fixed in the SN population. For HCN, markers S14_6050078 and S16_773999 showed associations with variation in cyanogenic potential. Overall, S1_24155522 emerged as the most promising validated candidate marker for MAS targeting carotenoid biofortification. However, the limited and population-dependent performance of markers associated with DMC and HCN indicates that these traits are governed by more complex genetic architectures and require further validation across diverse breeding populations and environments before routine deployment in marker-assisted selection. These findings provide valuable insights for improving the efficiency of molecular breeding strategies aimed at enhancing nutritional quality and food safety in cassava.

## 1 Introduction

Cassava (*Manihot esculenta* Crantz; *Euphorbiaceae*) is a starchy root crop of global socio-economic importance. Globally, over 1 billion people subsist on cassava as a staple food, a national food security, a source of foreign exchange and income through value-added products (FAO, 2017; Nyaika, 2024; Otekunrin, 2024). In Sub-Saharan Africa (SSA), cassava roots are utilized in the preparation of staple foods such as *gari*, *tapioca*, and *fufu* for human consumption, whereas the leaves are commonly used as nutritious fodder for livestock (Falace and Akingbala, 2010; Hossain et al., 2025; Iragaba et al., 2021). Furthermore, cassava constitutes a vital industrial raw material in the manufacture of adhesives, paper, beverages, and bioethanol, among others (Balagopalan, 2002). Global cassava production is estimated at approximately 333–342 million tonnes, harvested from about 30–34 million hectares, with average yield of ~10 t ha^-1^; West Africa, particularly Nigeria, contribute a major share based on recent FAOSAT data (Food and Agriculture Organization of the United Nations, 2025). Therefore, cassava’s multifaceted role in food systems, livelihoods, and industrial applications establishes it as a strategic crop for sustaining food security and driving economic development, particularly in Sub-Saharan Africa.

Despite cassava’s key role as a source of sustenance and important industrial raw material, its utilization for food and feed is hindered by several constraints in SSA. Some of these bottlenecks encompass the presence of high HCN content and micronutrient deficiency, among others (Lykkesfeldt and Lindberg Møller, 1994; Gleadow and Møller, 2014). Key quality traits influencing consumer acceptance include HCN content, DMC, and TCC, particularly in regions where cassava roots are boiled for consumption (Boakye-Peprah et al., 2020; Ige et al., 2022). High dietary intake of HCN may result in acute toxicity and fatality (Rivadeneyra-Domínguez and Rodríguez-Landa, 2020). In addition, prolonged exposure to sub-lethal doses of hydrogen cyanide (HCN) is associated with severe and irreversible neurological disorders, notably tropical ataxic neuropathy (TAN) (Kanaabi et al., 2024). Similarly, the deficiency of micronutrients in released cassava varieties, particularly vitamin A (VAD), exposes consumers to a range of health consequences associated with VAD, including measles, diarrhea, blindness, stunted growth, compromised immunity, higher early childhood mortality, and reproductive health complications in women, among others (Ashraf, 2025; Amoah et al., 2025). Among the health challenges corollary to VAD, blindness alone imposes an annual economic burden of approximately US$410.7 billion (PPP), ranging from US$322.1 billion to US$518.7 billion (PPP), worldwide (Bourne et al., 2021; Marques et al., 2021). As a result, cassava breeding efforts ought to focus on developing new varieties with enhanced micronutrient density through biofortification, coupled with low HCN and high DMC to improve the quality of life.

Conventionally, cassava breeding for improved root quality traits relies on phenotypic-based recurrent selection (PRS) (Ceballos et al., 2013; Garcia-Oliveira et al., 2020). Laboratory-based techniques such as High-Performance Liquid Chromatography (HPLC), iCheck fluorimeter (iCheck fluoro), and spectrophotometers are widely used for the quantification of total carotenoid content (TCC) in cassava breeding programs (Kimura et al., 2007; Andersson et al., 2017; Jaramillo et al., 2018). Comparative studies have shown that spectrophotometry provides the highest predictability for TCC, followed by iCheck and near-infrared spectroscopy methods, including desktop NIRS (dNIRS), while portable NIRS (pNIRS) tends to show lower repeatability and greater estimation bias (Jaramillo et al., 2018). In contrast, dry matter content (DMC) is commonly assessed using oven-drying or specific gravity methods (Wilhelm et al., 2004; Teye et al., 2011; Ikeogu et al., 2017; Silva et al., 2023), whereas hydrogen cyanide (HCN) is typically quantified using chemical assays (Bradbury et al., 1999; Fukuda et al., 2010; Kanaabi et al., 2024), reflecting the trait-specific nature of these measurement approaches. However, cassava breeding cycles can span nearly a decade due to factors such as long cropping durations (12–18 months), difficulties in hybridization, poor flowering and seed set, and low clonal multiplication rates, making conventional approaches inefficient and less reliable (Jennings and Iglesias, 2001; Ceballos et al., 2016; Ige et al., 2022; Kanaabi et al., 2024). Furthermore, the capital-intensive nature of biochemical methods for assessing quality traits such as TCC, DMC, and HCN underscores the need for alternative approaches, particularly the integration of molecular markers and other advanced breeding tools to accelerate selection and enhance genetic gain (Ceballos et al., 2015).

Marker-Assisted Selection (MAS) is a key molecular breeding approach that enables the precise identification and selection of desirable genotypes based on DNA-level information, which allows the selection of favorable alleles at trait-linked loci without direct phenotypic evaluation (Collard and Mackill, 2008; Parkes et al., 2015). In cassava, MAS facilitates early-stage evaluation of genotypes for root quality traits, overcoming the limitations of conventional breeding, which requires physiological maturity for trait assessment. Single Nucleotide Polymorphisms (SNPs) markers are increasingly recognized as the preferred genetic markers in MAS for crop improvement due to their numerous benefits and uniqueness (Collard and Mackill, 2008; Varshney, 2010). These include cost-effective genotyping, widespread presence across genomes, biallelic structure, and compatibility with high-throughput genotyping technologies (De Oliveira et al., 2012). This allows for detecting genetic polymorphisms among cassava genotypes, revealing useful alleles exploitable in cassava breeding (Rabbi et al., 2014a; Udoh et al., 2017). Udoh et al. (2017) utilized MAS in developing provitamin A-rich cassava by identifying and selecting alleles associated with high carotenoid content. Similarly, MAS has been successfully used to incorporate and pyramid multiple cassava mosaic disease (CMD) resistance genes from diverse genetic backgrounds into elite varieties, thereby enhancing the durability and breadth of resistance (OkogBenin et al., 2007, 2012; Thuy et al., 2021). Furthermore, MAS has been deployed in numerous cassava breeding programs to select genotypes harboring the favorable alleles for low HCN, high DMC, and abiotic stressors (Ceballos et al., 2015; Zou et al., 2017; Oliveira et al., 2018; Olasanmi and Kyallo, 2021; Esuma et al., 2022; Ige et al., 2022; Codjia et al., 2023). Thus, the deployment of MAS in cassava breeding could significantly shorten the breeding cycle and accelerate the development of new, improved varieties (Collard and Mackill, 2008).

Although MAS offers considerable advantages, its effectiveness depends largely on the availability of robust and validated markers associated with target traits. The identification of genomic regions or genes with significant association with traits of interest is essential for a successful deployment of MAS. The approaches for identifying genomic regions include GWAS and quantitative trait loci (QTL) mapping studies (Ioannidis, 2009; Vidya et al., 2021). Over the past decade, extensive studies have identified a large number of genomic regions associated with cassava root quality traits, pests, and diseases, and other abiotic stressors (Nzuki et al., 2017; Zhang et al., 2018; Garcia-Oliveira et al., 2020; Ewa et al., 2021; Phumichai et al., 2021; Hmwe et al., 2022; Olayinka et al., 2025; Liu et al., 2025; Bohorquez et al., 2025). Nonetheless, the identification of genes or QTL is just the first step towards the deployment of MAS. The identified QTLs must be validated in multiple genetic backgrounds and independent populations under the prevailing environmental conditions (Andelkovic et al., 2020; Ige et al., 2022).

The present study utilized nine trait-associated KASP markers distributed across three candidate genes, as previously reported by Udoh et al. (2017); Ogbonna et al. (2020a), and Rabbi et al. (2020). These markers are linked to key root quality and nutritional traits, including TCC, DMC, and HCN concentration. Consequently, this study was conducted to validate the effectiveness of nine SNP markers associated with dry matter content (DMC: S1_24197219, S6_20589894, S12_5524524), total carotenoid content (TCC: S1_24155522, S1_30543962, S5_3387558, S8_25598183), and hydrogen cyanide content (HCN: S16_773999, S14_6050078) across two cassava populations. We hypothesized that these nine SNP markers would be significantly associated with variation in TCC, DMC, and HCN across diverse cassava genetic backgrounds.

## 2 Materials and methods

### 2.1 Description of study population

A total of 435 cassava genotypes were evaluated in this study. These comprised 261 HarvestPlus Seedling Nursery (SN) progenies derived from 18 full-sib families developed from open-pollinated crosses among 23 progenitors of advanced breeding lines from the International Institute of Tropical Agriculture (IITA), Ibadan, Oyo State, Nigeria. The progenitors were selected for high carotenoid content, high dry matter, high starch, and resistance to Cassava Mosaic Disease (CMD). Additionally, 151 genotypes, including standard check varieties, from the University of Ibadan-derived clones (UIC) exhibiting variability in storage root color (ranging from white to deep yellow) were evaluated at the Advanced Yield Trial (AYT) stage. The UIC genotypes originated from yellow-root cassava varieties (IITA-TMS 1070593, IITA-TMS 1011371, IITA-TMS 1070539, IITA-TMS 1011368, and IITA-TMS-1011412), chosen for their high β-carotene content, CMD resistance/tolerance, and storage root yield released between 2011 and 2014. The 23 progenitors of the SN population were planted alongside the 261 progenies. The two populations were intentionally selected because they represent contrasting breeding stages and genetic backgrounds commonly used in cassava improvement programs, thereby providing an independent framework for validating previously identified trait-associated KASP markers rather than discovering novel marker–trait associations.

### 2.2 Study locations and experimental design

The field experiment was conducted during the 2020–2021 growing season at the International Institute of Tropical Agriculture (IITA) (7°24’ N, 3°54’ E; 200 m above sea level) and Department of Crop and Horticultural Sciences (7°27′N, 3°45′E), University of Ibadan, Ibadan, Oyo State, Nigeria. The SN population was evaluated at IITA using augmented experimental design with a spacing of 1.0 m between rows and 0.25 m within rows, where the cassava variety TMEB117 served as a repeated check across blocks to account for field variability. Botanical seeds for the SN were germinated in the greenhouse and transplanted as seedlings (20–25 cm tall) to the field, with harvesting conducted 8 months after planting (MAP). Concurrently, the UIC population was established on 12 May 2020 at the Teaching and Research Farm of the Department of Crop and Horticultural Sciences, University of Ibadan, Nigeria, using a randomized complete block design (RCBD) with two replications. Twenty stem cuttings were planted in each 20 m^2^ plot with 1.0 m × 1.0 m, with standard check varieties included in each block. Storage roots were harvested 12 months after planting (MAP). Standard and technical recommendations for cassava were followed for field management practices at both experimental stations.

### 2.3 Genotyping

Genotyping was conducted on a subset of 360 genotypes selected from the overall trial populations. The subset was chosen to maximize the availability of complete phenotypic records while ensuring adequate representation of the genetic and phenotypic diversity present in both populations. This included all 261 SN progenies, 76 AYT genotypes selected from the initial 151 based on availability of phenotypic data and representation of carotenoid variation, and the 23 parental genotypes used in developing the SN population.

Leaf samples from juvenile plants in both populations were collected by obtaining three leaf discs (6 mm diameter) per plant. The discs were placed in 96-deep well plates on ice, covered with paraffin oil, and freeze-dried at the Bioscience Center, IITA, using a LABCONCO FreeZone 18 L −50 °C freeze-dryer at −51 °C and 5.0 pas for at least 72 hours until completely dry. The freeze-dried samples were subsequently shipped to Intertek Laboratory, Australia, for genotyping using nine trait-associated KASP markers distributed across three candidate genes, as reported in previous studies (Udoh et al., 2017; Ogbonna et al., 2020a; Rabbi et al., 2020; Anokye et al., 2026) (see **Tables 1**, **2**). These markers targeted traits related to provitamin A carotenoid, DMC, and HCN (**Table 1**). The KASP assay allowed precise bi-allelic discrimination of SNPs using a fluorescence resonance energy transfer quencher cassette, ensuring accurate detection of known SNPs within the cassava genotypes. Two non-template controls (NTC) were included in each plate, and all reactions were conducted following the KASP genotyping manual (LGC Genomics, 2013). Negative controls were included to monitor contamination and ensure the reliability of genotype calling throughout the genotyping process.

**Table 1:** Description of nine trait-linked KASP markers used for genotyping in the two populations.

Traits	SNP name	LG	Favorable allele	Unfavorable allele	Reference
Dry matter content	S1_24197219		C	T	Rabbi et al. (2020)
	S6_20589894		G	A	Rabbi et al. (2020)
	S12_5524524		C	T	Rabbi et al. (2020)
Carotenoid Content	S1_24155522	*PSY2*	A	C	Udoh et al. (2017)
	S1_30543962		G	A	Rabbi et al. (2020)
	S5_3387558		T	C	Rabbi et al. (2020)
	S8_25598183		T	G	Rabbi et al. (2020)
Hydrogen cyanide	S16_773999		A	G	Ogbonna et al. (2020a)
	S14_6050078		G	A	Ogbonna et al. (2020a)

LG, Linkage group, *PSY2*: *Phytoene synthase* 2.

**Table 2:** Descriptive statistics of two carotenoid content measures, dry matter, and hydrogen cyanide contents in the advanced yield trial (AYT).

Trait	N	mean	min	max	MS	σ^2g^	σ^2e^	*H^2^*
iCheck (µg g^−1^)	73	5.85	1.14	14.40	6.56	6.14	0.41	0.94
TCC_chart	151	1.88	1.00	5.00	0.98	0.86	0.13	0.87
DMC (%)	151	23.52	7.59	43.29	26.51	3.12	20.27	0.13
HCN (mg 100 g^−1^)	151	24.53	3.85	71.76	131.50	86.70	44.80	0.66

σ^2g^, genotypic variance; σ^2e^, residual variance; *H^2^*, Within-trial broad-sense heritability; N, Sample size; TCC_chart, Total carotenoid or color chart. The smaller sample size for TCC (iCheck Fluoro) reflects the subset of genotypes for which fluorometric measurements were available.

Genotyping was performed using high-throughput PCR-based SNPline workflow in 384-well plates with a reaction volume of 1µL per well. Each KASP genotyping reaction consisted of approximately 10 ng of genomic DNA, a marker-specific assay containing allele-specific primers, and KASP-TF™ Master Mix. The master mix included fluorescence resonance energy transfer (FRET)-based reporters (FAM and HEX), a passive reference dye (ROX™), Taq DNA polymerase, deoxynucleotide triphosphates (dNTPs), MgCl_2_, and an optimized reaction buffer. For each SNP, the assay comprised two allele-specific forward and common reverse primers. Following PCR amplification, fluorescence signals were measured, and genotype calls were assigned using KRAKEN™ software based on the separation of allele-specific fluorescence clusters (**Supplementary Table 6**). Samples producing clear and unambiguous genotype clusters were assigned as homozygous, whereas samples with poor amplification or ambiguous fluorescence signals were classified as missing data. Missing genotype calls were excluded from marker-specific analysis but were not imputed.

### 2.4 Phenotyping and data collection

Genotypes in both populations underwent plant vigor assessment three months after planting and were evaluated for storage root yellowness, TCC, DMC, and HCN content at harvest. Harvesting occurred 8 and 12 months after planting (MAP) in the SN trial and AYT. Previous reports have shown that carotenoid concentration and dry matter content remain relatively stable after eight months of growth, indicating that harvested at either 8 or 12 months provides comparable estimates of these traits for breeding evaluations (Hue et al., 2012). Consequently, the different harvest ages used for the SN and AYT populations were considered appropriate for marker validation while reflecting their respective breeding stages. Phenotypic data from both populations were integrated with KASP genotyping data to biologically validate previously reported SNP markers associated with TCC, DMC, and HCN.

#### 2.4.1 Determination of TCC, DMC, and HCN

Total carotenoid content (TCC), dry matter content (DMC), and hydrogen cyanide (HCN) were quantified using established laboratory protocols for each experimental population. Roots were harvested, peeled, diced, mixed, and divided into three portions for analysis. The DMC was determined after harvest, as described by Esuma et al. (2016) (Equation 1). To determine the dry matter content (DMC), approximately 100 grams of homogenized tissue per genotype were oven-dried at 105 °C for 24 hours until a constant weight was achieved. The dried material was then weighed to obtain dry weight, and DMC was computed using the following formula.

(1) $$\text{DMC }(\%)\;=\frac{\text{DSW}}{\text{FSW}}\;\times 100$$

Where DSW = dry sample weight and FSW = fresh sample weight.

Hydrogen cyanide (HCN) concentration was determined using automated enzymatic method with an Auto-Analyser (Technicon AA II) following Rao and Hahn (1984). Laboratory analyses were subsequently conducted at the iCheck and Food Science and Nutrition Sciences laboratories, IITA, Ibadan, Nigeria. Cassava storage roots were taken to the food science lab (IITA) for extraction immediately after the harvest. Healthy-looking storage roots were selected and treated as a single sample for each genotype. The whole storage roots were peeled, diced, and mixed thoroughly, and 50 g were immediately transferred to bottles with screw caps containing 160 ml of extracted solution (orthophosphoric acid, 0.1M).

The samples were homogenized using an electric blender and subsequently centrifuged at 13, 000 rpm. For cyanide analysis, some extracts were processed immediately, while others were refrigerated overnight at 4 °C for later assessment. All laboratory analyses were performed following protocols routinely employed at IITA to ensure consistency and comparability among genotypes.

Cold orthophosphoric acid was used for extraction, followed by the addition of exogenous linamarase to catalyze the release of cyanide ions. These ions reacted with chloramines T, isonicotinate, and dimethyl barbiturate to form a colored complex, the intensity of which correlated with the cyanide concentration and was quantified as mg HCN per 100 grams of sample. To release cyanide bound in complex forms, samples were exposed to ultraviolet light under acidic conditions. The treated solution was then passed through a dialyzer, where acidification enabled the formation of free cyanide ions. These ions diffused across a membrane into an alkaline stream, where cyanide concentration was measured amperometrically using a silver working electrode alongside a silver/silver chloride reference electrode (Rao and Hahn, 1983).

Carotenoid content was assessed using two methods: (i) indirect visual estimation based on root parenchyma color using a CIAT-developed color chart (scale 1–9; 1 = white to 9 = pinkish) (Esuma et al., 2016), and (ii) direct quantification using the iCheck Fluorometer (BioAnalyt GmbH, Teltow, Germany). Total carotenoid content was extracted using acetone with a mortar and pestle following Rodriguez-Amaya et al. (2004) Equation 2. Total carotenoid content (TCC) was calculated as:

(2) $$\text{TCC }(\text{µ}\frac{\text{g}}{\text{g}})=\frac{\text{Vs}}{\text{WS}}\;\times \text{A}*DF$$

Where Vs is the volume of solution transferred to the Falcon tube, Ws is the fresh weight of the sample, A is the absorbance of the iEx™ CAROTENE vial at a wavelength of 450 nm, and *DF* is the dilution factor (total sample volume in water divided by sample weight) as described by Esuma et al. (2016). All procedures were conducted under dark conditions to minimize light-induced degradation.

### 2.5 Statistical analysis

#### 2.5.1 Phenotypic and laboratory data analysis

The data were analyzed using R Statistical Software version 4.1 (R Development Core Team, 2020). Histograms were used to visually assess the distribution of TCC, DMC, and HCN in each population. Formal tests of normality were not performed because the mixed-effects models used are robust to moderate deviations from normality and the large sample sizes provide reliable parameter estimation. using raw data from both trials. For the Advanced Yield Trial (AYT), a linear mixed-effects model was fitted using restricted maximum likelihood (REML) to estimate best linear unbiased estimates (BLUEs) for all entries. The AYT consisted of 151 genotypes, including standard check varieties evaluated alongside test entries. All genotypes (including checks) were treated as fixed effects, while blocks and replicates were modeled as random effects, consistent with a randomized complete block design (RCBD). The model was implemented using the lme4 package in R (Bates et al., 2015; R Development Core Team, 2020). The mixed-effects model was used to account for the experimental design and to obtain adjusted genotype means (BLUEs) for subsequent analyses, including estimation of heritability and marker validation (Equation 3). The mathematical model (Kling, 2011) below was used:

(3) $${\text{Y}}_{\text{ij}}\;=\;µ\;+\;{\text{G}}_{\text{i}}+{\text{ β}}_{\text{j}}\;+\;{\text{ϵ}}_{\text{ij}}$$

Where Y_ij_ is the observed phenotypic value of the *i^th^* genotype in the *j^th^* block, µ is the overall mean, G_j_ is the fixed effect of the i^th^ genotype (including checks), ${\text{β}}_{\text{j}}$ is the random effect of the *j^th^* block, and *j*_ij_ is the residual error.

Pairwise Pearson correlation coefficients were calculated using corr*.test* function in the psych package to quantify the relationships among TCC, DMC, and HCN within each population (R Development Core Team, 2020). Within-trial broad-sense heritability (H^2^) was estimated from variance components obtained from the mixed-effects model using the BLUEs of the AYTY population (**Supplementary Table 5**) (Equation 4). The estimate represents the proportion of phenotypic variance attributable to genetic differences among genotypes within the experimental environment and was calculated as:

(4) $${\text{H}}^{2}\;=\;\frac{{\text{σ}}^{2}\text{g}}{{\text{σ}}^{2}\text{g }+{\text{ σ}}^{2}\text{e}}$$

Here, H^2^ is the within-trial broad-sense heritability, while σ^2^g and σ^2^e denote the variance components attributed to genotypic effects and residual error, respectively. This estimate was used to assess the relative contribution of genetic effects to phenotypic variation under the experimental conditions and to evaluate the potential effectiveness of phenotypic selection for each trait.

#### 2.5.2 Technical and biological validation of KASP markers

The robustness of SNP markers was evaluated using technical performance metrics, including call rate and call clarity. KASP fluorescence data were analyzed using SNPviewer™ software (LGC Genomics, UK) to assign genotype calls and visualize allelic clusters based on FAM and HEX signals. Samples were classified into homozygous and heterozygous genotypes, and cluster quality was assessed to ensure reliable genotyping. Call rate was defined as the proportion of samples for which genotype calls were successfully obtained, excluding missing data. Call clarity referred to the distinctness of genotype clustering on the fluorescence-based Cartesian plots, where well-separated and compact clusters indicate reliable discrimination among genotype classes. For biallelic SNPs in a diploid genome, this corresponds to clear differentiation of three expected genotypic groups: homozygous for allele 1, homozygous for allele 2, and heterozygous.

Biological validation of the markers was conducted using complementary analytical approaches. Because this study focused on validating a small set of previously reported candidate KASP markers (**Table 1**), rather than identifying novel marker–trait associations through genome-wide discovery, all statistical tests were performed within a hypothesis-driven validation framework. Accordingly, statistical significance was evaluated at α = 0.05 without adjustment for genome-wide multiple testing. First, allele substitution effects were evaluated using boxplots, and differences in best linear unbiased estimates (BLUEs) for TCC, DMC, and HCN among marker genotype classes were tested using a pairwise *t-test*. For boxplot analysis, 259 of the 261 genotyped individuals were included, while 2 individuals were excluded because of missing genotype data. Boxplots were generated using ggplot2 in R software version 4.1 to visualize the distribution of phenotypic values among marker genotype classes and to illustrate marker substitution effects (R Development Core Team, 2020).Pairwise comparisons among marker genotype classes were performed using the pairwise_t_test function in the rstatix package in R at a significance level of α = 0.05.

Second, the predictive performance of the SNP markers was assessed using a multiple linear regression model, in which marker genotypes were treated as predictor variables and observed phenotypic values as the response variable (Equation 5). The regression analysis was performed to evaluate the predictive ability of the validated marker set rather than to infer causal genetic effects.

(5) $$Y=\;µ+\;{µ}_{1}+\;\;{µ}_{2\;}+\ldots \;{µ}_{n}+e$$

Where: Y = phenotypic observations of the traits, µ = overall mean of the population, µ_1,_ µ_2,_ µ_3_ = marker effects, e = residual value.

To quantify the contribution of individual SNP markers to phenotypic variation, the percentage of variance explained (PVE) was estimated from analysis of variance (ANOVA) outputs. PVE was estimated separately for each marker to quantify its individual contribution to phenotypic variation explained by that marker and therefore complements, rather than replaces, the within-trial broad-sense heritability estimates obtained from the mixed-effects model (Equation 6). For each marker, the sum of squares (SS) was computed as the product of the mean square (MS) and the corresponding degrees of freedom (df). The PVE for each marker was then calculated as the ratio of marker SS to the total phenotypic SS, expressed as:

(6) $$PVE\;(\%)=\;\frac{SS_{marker}}{SS_{total}}\;\times 100$$

Where SS_marker_ is the sum of squares attributed to the SNP marker, and SS_total_ represents the total phenotypic sum of squares of the marker and residual SS. Residual SS, representing unexplained variation, was derived from the error term of the ANOVA model and incorporated into the total SS for each trait and population. This approach enabled the quantification of the contribution of each validated SNP marker to phenotypic variation in total carotenoid content (TCC), dry matter content (DMC), and hydrogen cyanide (HCN) across both cassava populations.

## 3 Results

### 3.1 Phenotypic variation, heritability and traits relationships

**Figure 1:**
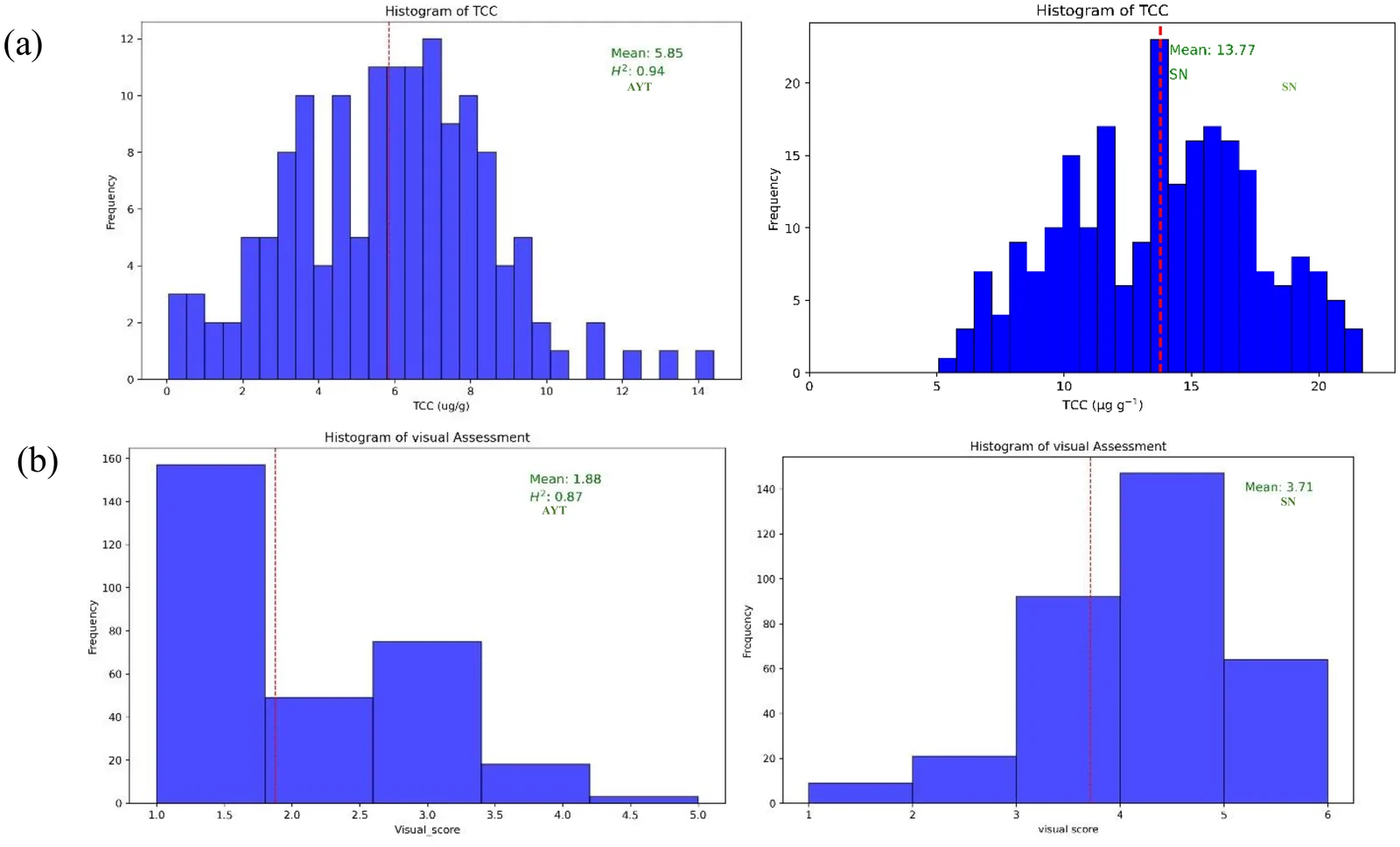
Distribution of carotenoid concentration as quantified by iCheck fluorimeter **(a)** and visual color assessment by color chart **(b)** in the two populations: Advanced Yield Trial (AYT) and Seedling Nursery population (SN). The histograms show the frequency distribution of measurements within each population. The red dashed vertical line represents the mean value of the trait for the respective population. Values of mean and Within-trial broad-sense heritability (H²) (for AYT only) are indicated in each panel.

Substantial phenotypic variation was observed for total carotenoid content (TCC), dry matter content (DMC), and hydrogen cyanide (HCN) across the Advanced Yield Trial (AYT) and Seedling Nursery (SN) populations. Total carotenoid content using the iCheck fluorimeter exhibited an approximately normal frequency distribution in both populations, with mean values of 13.77 µg g^−1^ (SN) and ranged from 5.08 to 21.71 µg g^−1^ (**Figure 1a**). In the AYT population, the mean TCC was 5.85 µg g^−1^, with values ranging from 1.14 to 14.40 µg g^−1^ (**Figure 1b**). Visual assessment using the color chart showed a near-normal distribution in the SN population, whereas a positively skewed distribution was observed in the AYT population, with mean scores of 3.71 and 1.88, respectively. Color chart scores ranged from 2 to 6 in the SN population and from 1 to 5 in the AYT population.

**Figure 2:**
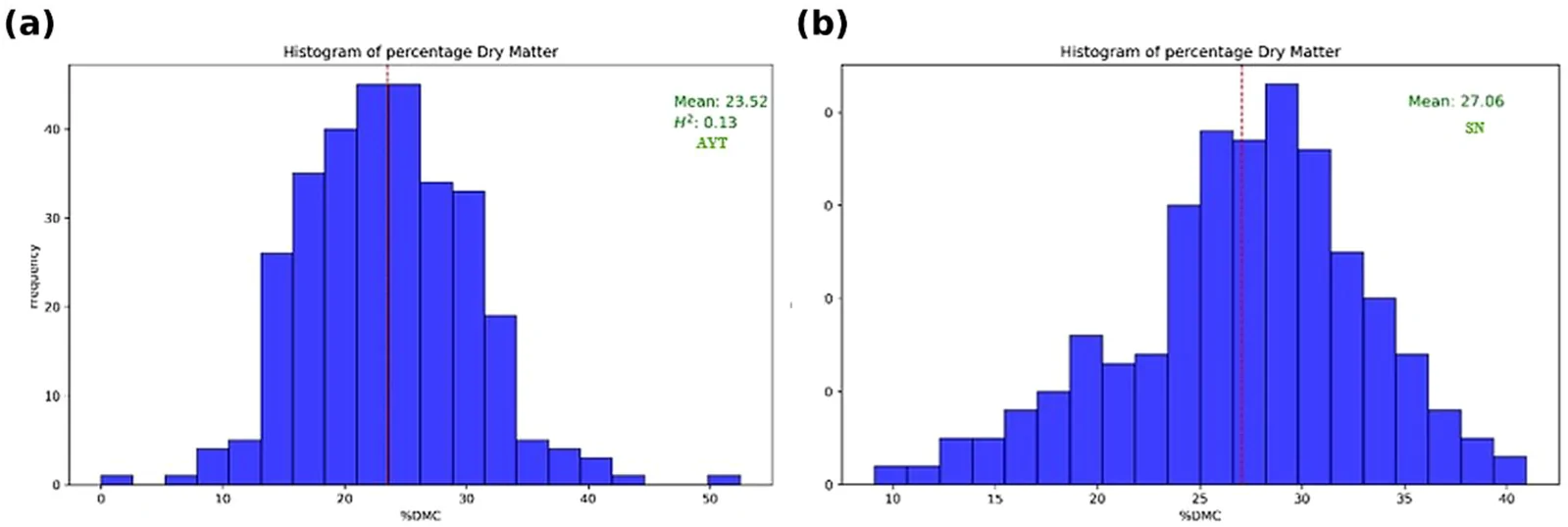
Distribution of dry matter content (DMC) in the two cassava populations. **(a)** Advanced Yield Trial (AYT) population and **(b)** Seedling Nursery (SN) population. Histograms show the frequency distribution of DMC measurements within each population. The red dashed vertical line represents the population mean. Mean values and within-trial broad-sense heritability (H^2^; AYT only) are indicated in each panel.

The phenotypic variability of DMC among the genotypes across the two populations showed continuous variation, with mean values of 23.52% in the AYT and 27.05% in the SN. The observed ranges were 7.59-43.29% in AYT and 5.0-40.9% in the SN (**Figure 2**). In the AYT population, DMC generally exhibited an inverse relationship with TCC. In contrast, a positive association was observed in the SN population, suggesting population-specific relationships between these traits. A subset of genotypes exhibited relatively high DMC together with high TCC. The continuous distribution observed for both DMC and TCC was consistent with their quantitative inheritance.

**Figure 3:**
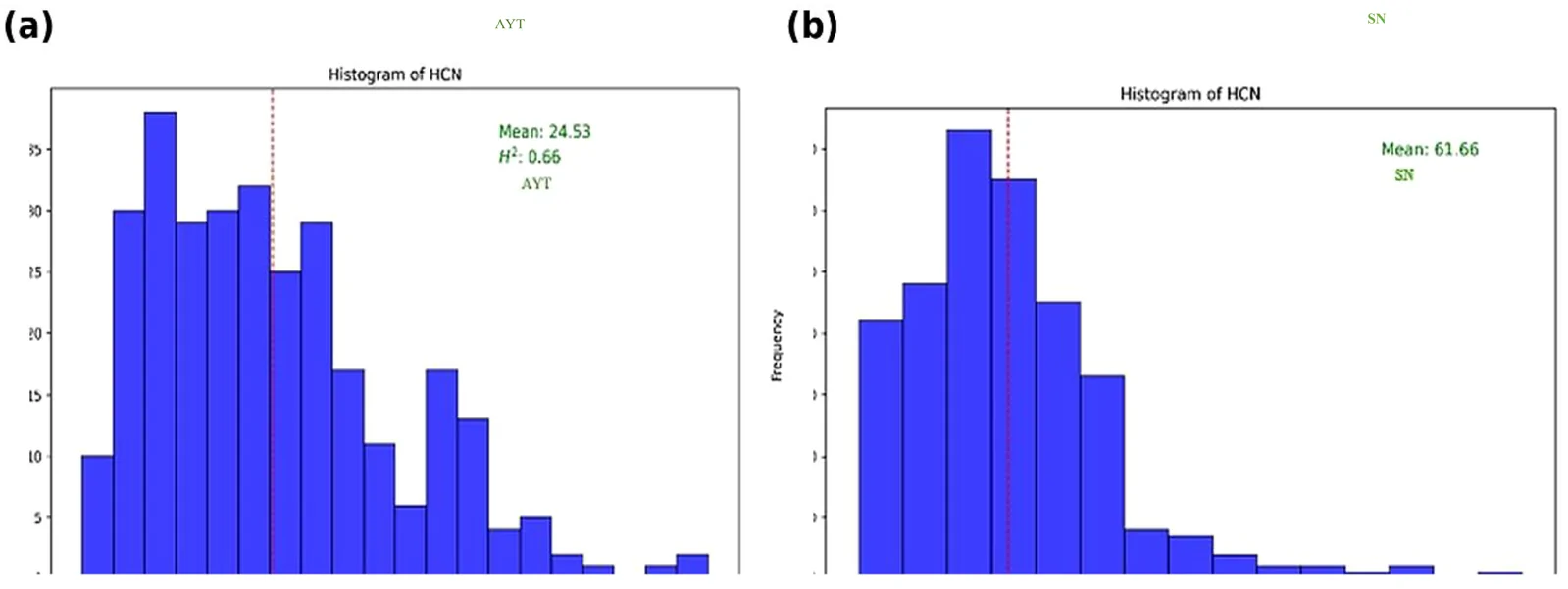
Distribution of hydrogen cyanide (HCN) content in the two cassava populations. **(a)** Advanced Yield Trial (AYT) population and **(b)** Seedling Nursery (SN) population. Histograms show the frequency distribution of HCN measurements within each population. The red dashed vertical line represents the population mean. Mean values and within-trial broad-sense heritability (H^2^; AYT only) are indicated in each panel.

The distribution of hydrogen cyanide (HCN) (**Figure 3**) exhibited positive skewness, with HCN content ranging from 3.85 to 71.8 mg 100 g^−1^ in the AYT and from 10.5 to 257.0 mg 100 g^−1^ in the SN trials, with average values of 24.53 mg 100 g^−1^ and 61.66 mg 100 g^−1^, respectively.

Seven families in the SN trial (IBASN201076, IBASN201098, IBASN201125, IBASN201144, IBASN201149, IBASN201156, and IBASN201160) had mean total carotenoid content (TCC) values (color chart) ≥5 and mean iCheck Fluoro values ranging from 13.64 to 14.77 µg g^−1^. Across these families, mean dry matter content (DMC) ranged from 26.04 to 31.08%, while mean hydrogen cyanide (HCN) content ranged from 45.75 to 92.23 mg 100 g^−1^ (**Supplementary Table 4**). These results demonstrate variation among families for TCC, DMC, and HCN, providing useful information for selecting parental materials in cassava breeding programs.

Within-trial broad-sense heritability estimates varied among traits. TCC showed high heritability (H² = 0.94 for iCheck; 0.87 for visual score), while HCN exhibited moderate to high heritability (H² = 0.66). In contrast, DMC had a low heritability (H² = 0.13), indicating comparatively greater environmental influence on phenotypic expression (**Table 2**).

**Figure 4:**
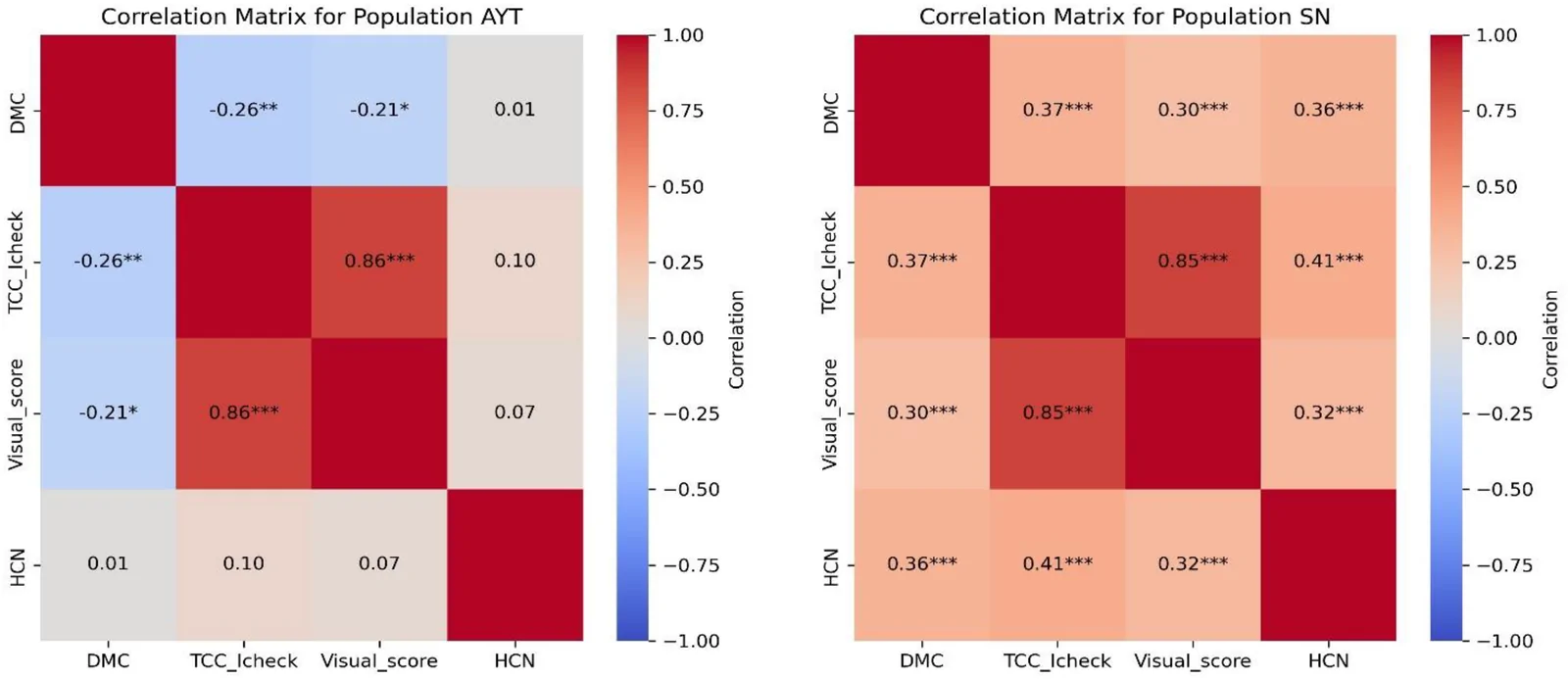
Pairwise correlation matrix showing the relationship among total carotenoid content (TCC) as measured by iCheck fluorimeter device (TCC_icheck), dry matter content (DMC), hydrogen cyanide (HCN), and visual root color score in the Advanced Yield Trial (AYT; left) and Seedling Nursery (SN; right) population. Color intensity represents the strength and direction of correlations, where red indicates positive correlation, blue indicates negative correlation, and color intensity reflects the magnitude of the correlation coefficient (stronger colors indicate stronger relationships). Asterisks denote statistically significant correlation (p < 0.05, p < 0.01, *p < 0.001).*.

Pearson’s correlation coefficients among the studied traits in AYT and SN populations are presented in **Figures 4a, b**. In the AYT population, DMC showed weak negative correlations with TCC measured by iCheck Fluoro (r = −0.26) and visual score (r = −0.21), and a negligible but positive correlation with HCN (r = 0.01). TCC measured by iCheck Fluoro exhibited a strong positive correlation with visual score (r = 0.86) and a weak positive correlation with HCN (r = 0.10), while visual score showed a weak positive correlation with HCN (r = 0.07).

In the SN population, DMC exhibited moderate positive correlations with TCC measured by iCheck Fluoro (r = 0.37), visual score (r = 0.30), and HCN (r = 0.36). TCC measured by iCheck Fluoro showed a strong positive correlation with visual score (r = 0.85) and a moderate positive correlation with HCN (r = 0.41), while visual score exhibited a moderate positive correlation with HCN (r = 0.32).

### 3.2 Technical validation of KASP assays

#### 3.2.1 SNP call rate, clarity, and genotypic distribution

High call rates (96-100%) were consistently obtained across the AYT, SN, and parental populations, indicating excellent technical performance of the KASP assays. Genotyping samples across the two populations and the progenitors (**Supplementary Figures 1**–**3**) showed clear and well-defined genotype clusters for the nine trait-linked Single Nucleotide Polymorphism (SNP) markers. Three distinct groups, representing favorable homozygotes, heterozygotes, and unfavorable homozygote clusters, were discernible for all markers except for S1_24155522, S1_24197219, and S12_5524524, based on the fluorescence profile of the Kompetitive Allele-Specific PCR (KASP) assays. Specifically, markers S1_24155522 and S1_24197219 distinguished only the homozygote alleles (AA and TT, respectively) among the Seedling Nursery (SN) and the progenitors **Supplementary Figures 1**, **2**. Meanwhile, marker S12_5524524 identified only one homozygote (TT) and the heterozygote (CT) within the Advanced Yield Trial (AYT) (**Supplementary Figure 2**).

In the Advanced Yield Trial (AYT) and the Seedling Nursery (SN) trials, genotypic frequencies for marker S1_24155522 were 68.4% (AA), 21.1% (CA), and 4.0% (CC) in the AYT, and 100% (AA), 0% (CA), and 0% (CC) in the SN, with 6.5% uncallable in the AYT (**Supplementary Table 1**). For marker S1_30513962, genotypes were distributed as 10.5% (GG), 61.8% (GA), and 26.4% (AA) in the AYT, and 11.2% (GG), 52.3% (GA), and 36.4% (AA) in the SN. Marker S5_3387558 showed 1.3% (TT), 86.8% (TC), and 11.8% (CC) in the AYT and 72.6% (TC) and 27.4% (CC) in the SN. For S8_25598183, genotypes were 57.5% (GG), 36.9% (TG), and 5.5% (TT) in the AYT, and 27.8% (GG), 57.1% (TG), and 14.7% (TT) in the SN. Marker S1_24197219 exhibited a high frequency of unfavorable homozygotes, representing 79.0% of the AYT population and all individuals in the SN Population. Marker S6_20589894 showed a higher proportion of heterozygous genotype (AYT = 35.59%, SN = 52.9%).

Conversely, marker S12_5524524 predicted 70% of genotypes having favorable alleles and 58.3% heterozygotes in the AYT and SN trials, respectively. Genotypes bearing a single or double copy of the reference allele co-segregated for the three DMC markers with high dry matter content in both populations. The two markers (S16_773999 and S14_6050078) associated with a low level of hydrogen cyanide (HCN) both identified a relatively low proportion of genotypes (10.9% and 2.7% for AYT; 37.2% and 5% for SN) with a favorable allele in the two populations. Heterozygotes were higher in both populations except marker S16_773999, which had fewer (20.9%) in the SN trial.

A substantial proportion of individuals in both populations exhibited homozygosity for the unfavorable alleles GG (marker S16_773999) and AA (marker S14_6050078). Conversely, genotypes carrying two copies of the favorable allele demonstrated lower hydrogen cyanide (HCN) concentrations than heterozygous and unfavorable homozygous genotypes. Overall, the observed genotype distributions were consistent with the corresponding phenotypic variation for HCN across both populations.

### 3.3 Biological validation of KASP assays

#### 3.3.1 Allelic substitution effects

**Figure 5:**
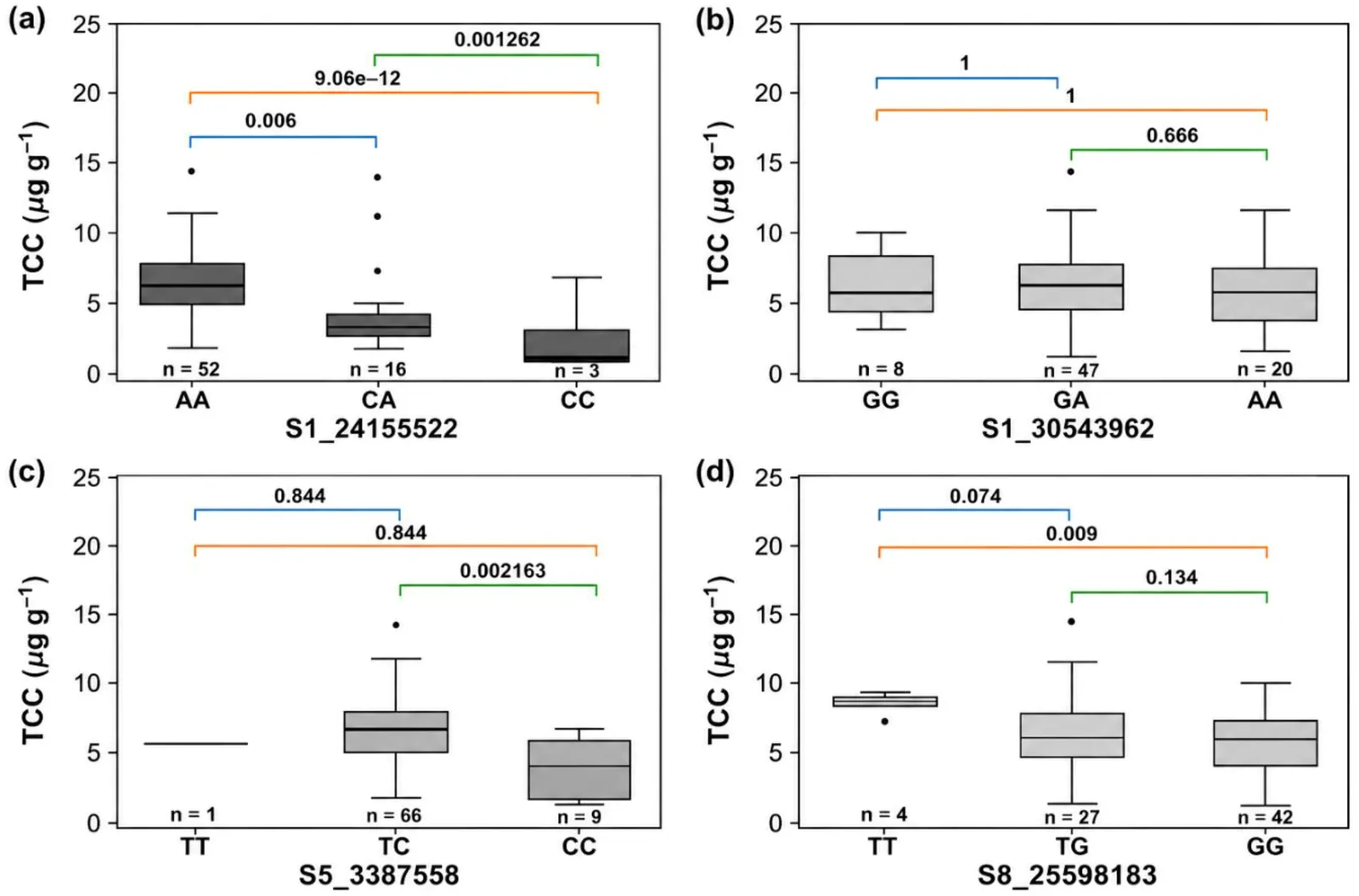
Allelic substitution effects of KASP markers associated with total carotenoid content (TCC) in the Advanced Yield Trial (AYT) population. **(a)** S1_24155522, **(b)** S1_30543962, **(c)** S5_3387558, and **(d)** S8_25598183. Boxplots show the distribution of TCC among marker genotype classes. Numbers below each box indicate sample size (n), and brackets denote pairwise comparisons with corresponding P values.

**Figure 6:**
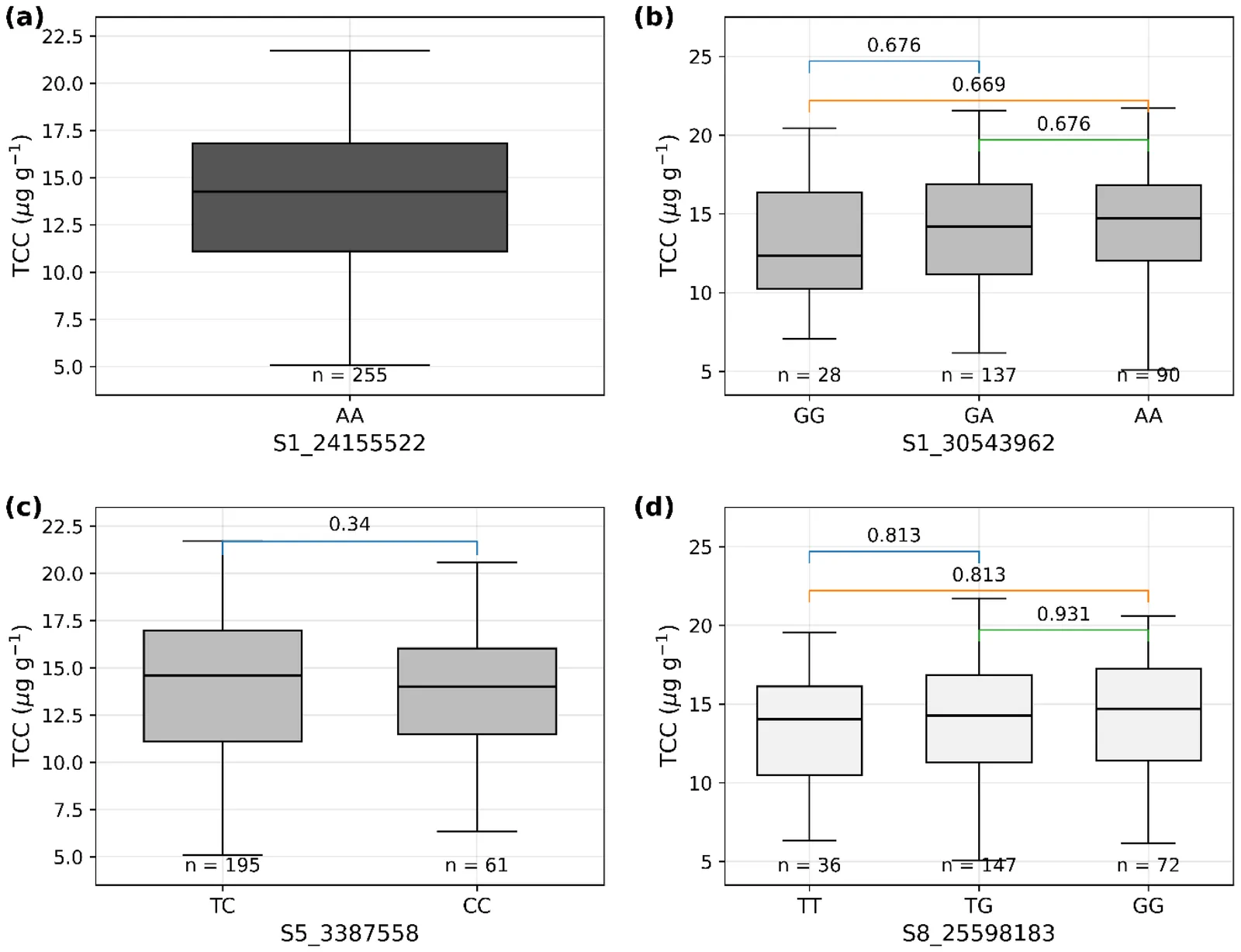
Allelic substitution effects of KASP markers associated with total carotenoid content (TCC) in the Seedling Nursery (SN) population. **(a)** S1_24155522, **(b)** S1_30543962, **(c)** S5_3387558, and **(d)** S8_25598183. Boxplots show the distribution of TCC among marker genotype classes. Numbers below each box indicate sample size (n), and brackets denote pairwise comparisons with corresponding P values.

**Figure 7:**
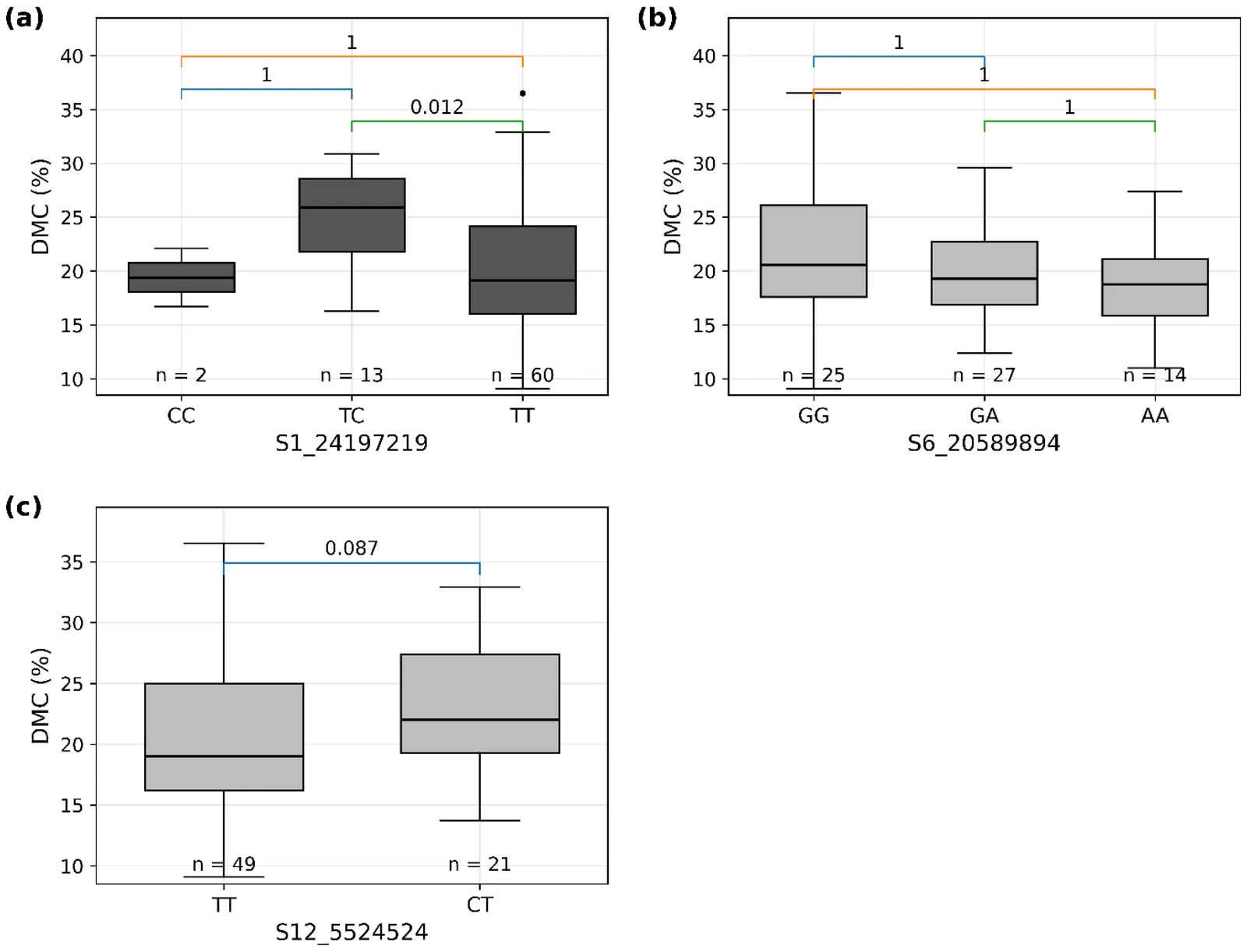
Allelic substitution effects of KASP markers linked to dry matter content (DMC) in the Advanced Yield Trial (AYT) population. **(a)** S1_24197219, **(b)** S6_20589894, and (c) S12_5524524. Boxplots show the distribution of DMC among marker genotype classes. Numbers below each box indicate sample size (n), and brackets denote pairwise comparisons with corresponding P values.

**Figure 8:**
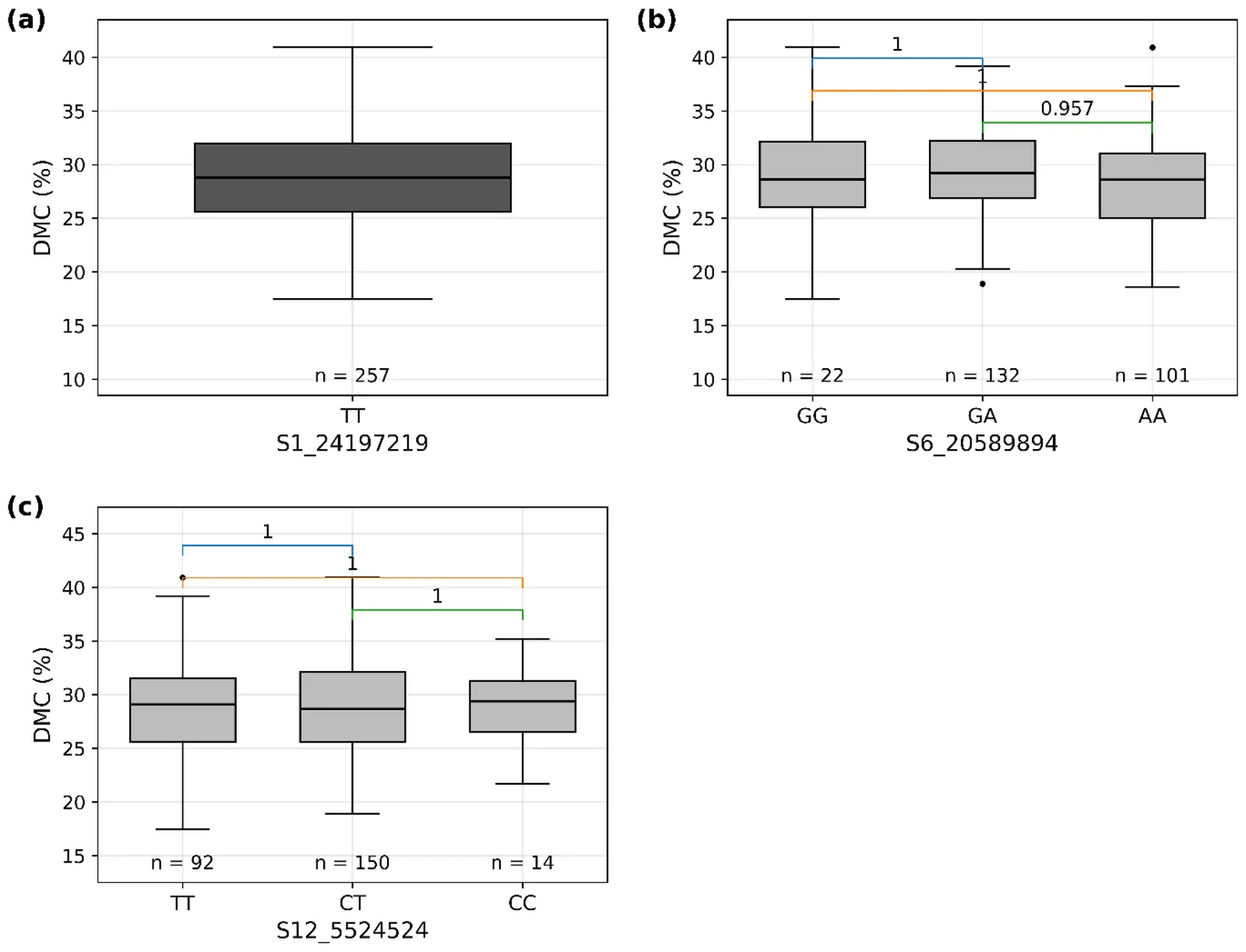
Allelic substitution effects of KASP markers linked to dry matter content (DMC) in the Seedling Nursery (SN) population. **(a)** S1_24197219, **(b)** S6_20589894, and **(c)** S12_5524524. Boxplots show the distribution of DMC among marker genotype classes. Numbers below each box indicate sample size (n), and brackets denote pairwise comparisons with corresponding P values.

**Figure 9:**
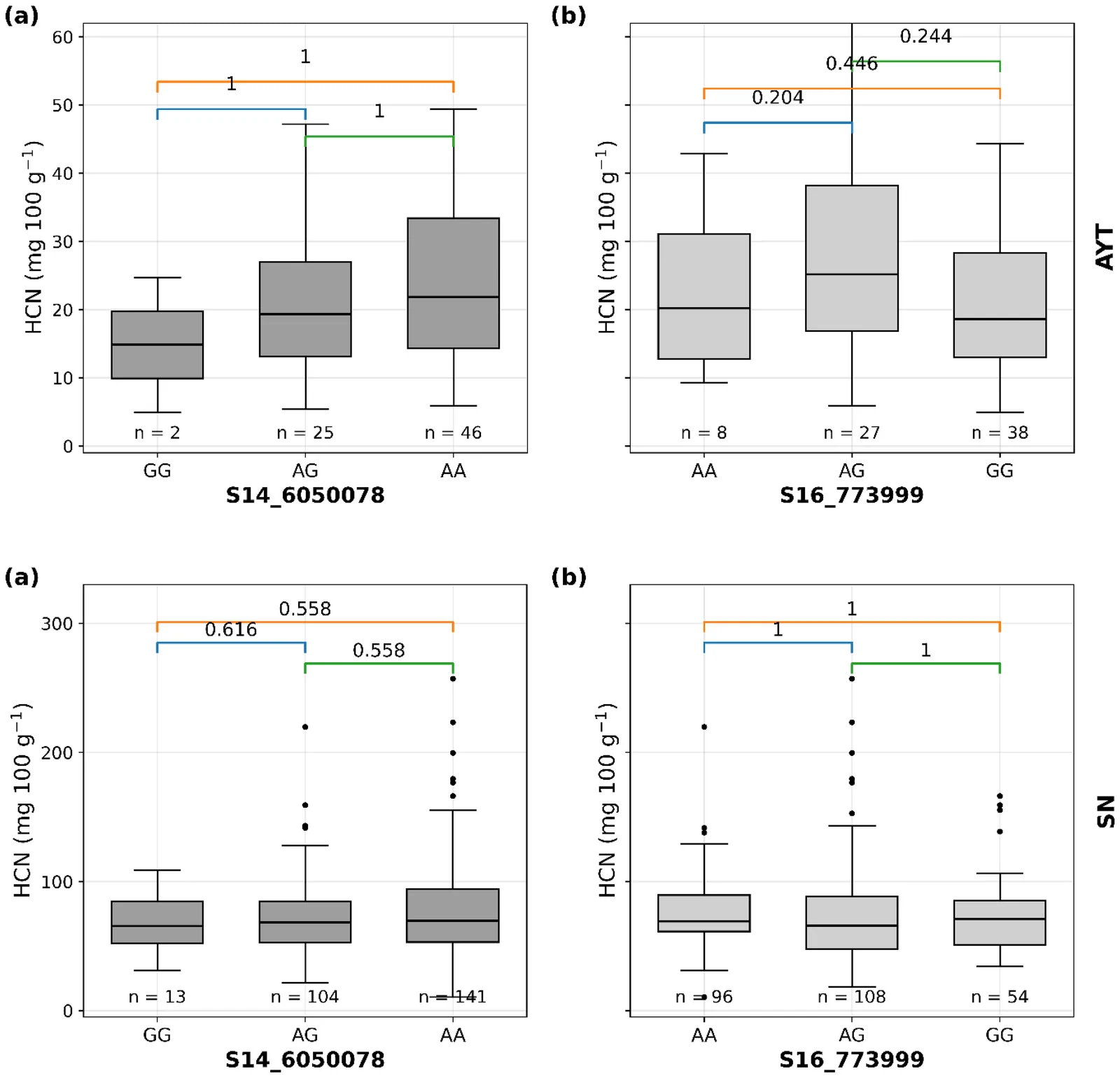
Allelic substitution effects of KASP markers associated with hydrogen cyanide (HCN) content in the Advanced Yield Trial (AYT) and Seedling Nursery (SN) populations. **(a)** S14_6050078 in the Advanced Yield Trial (AYT) population, **(b)** S16_773999 in the AYT population, **(c)** S14_6050078 in the Seedling Nursery (SN) population, and **(d)** S16_773999 in the SN population. Boxplots show the distribution of HCN content among marker genotype classes. Numbers below each box indicate sample size (n), and brackets denote pairwise comparisons with corresponding P values.

The boxplots depict allelic substitution effects for the nine trait-linked markers including genotypes from Advanced Yield Trial (AYT) and genotypes from Seedling Nursery (SN) populations. Marker effects were evaluated for total carotenoid content (TCC), dry matter content (DMC), and hydrogen cyanide (HCN) (**Figures 5**–**9**). The favorable alleles at markers S1_24155522, S1_30543962, S5_3387558, and S8_25598183 were A, G, T, and T, respectively. In the AYT population, pairwise differences in TCC were observed among AA, CA, and CC genotype classes at marker S1_24155522 (**Figure 5a**), with AA genotype exhibiting the highest TCC values (p < 0.001). Likewise, for SNP S5_3387558 (**Figure 5c**), significant differences were observed between TC and CC genotypes (p = 0.002), while SNP S8_25598183 (**Figure 5d**) showed marginal significance between TG and GG genotypes (p = 0.009). However, markers S1_30543962 (**Figure 5b**) exhibited no significant differences in TCC across the genotypes (GG, GA, AA) (p = 1.0).

Genotype-class sizes varied among markers, particularly for some favorable homozygous classes (e.g., TT for S5_3387558). Several genotype classes contained relatively few individuals because of low allele frequencies or fixation within the study populations; therefore, statistical comparisons involving these genotype classes should be interpreted with caution. This was particularly evident for markers S5_3387558 and S8_25598183 in the AYT population, where one favorable homozygous genotype class contained only 1 and 3 individuals, respectively.

In the SN population, all individuals carried the AA genotype at marker S1_24155522, indicating complete fixation at this locus and precluding comparisons among genotype classes (**Figure 6a**). Although substantial phenotypic variation in TCC was observed among these individuals, genotype-class comparisons were not possible because only one genotype was present. Two genotype classes, TC and CC, were observed at marker S5_3387558 (**Figure 6c**), whereas three genotype classes were observed at markers S1_30543962 and S8_25598183 (**Figures 6b, d**). No significant differences in TCC were detected among genotype classes for S1_30543962, S5_3387558, or S8_25598183 in the SN population (p > 0.05). For dry matter content (DMC), the favorable allele at markers S1_24197219, S6_20589894, and S12_5524524 were C, G, and C, respectively (**Figures 7**, **8**). For marker S1_24197219 (**Figure 7a**), a significant difference in DMC was detected between the TC and TT genotype classes (p = 0.012), with the TC genotype exhibiting the highest DMC values. No significant differences in DMC were observed among genotype classes for markers S6_20589894 (**Figure 7b**) and S12_5524524 (**Figure 7c**). Genotype class sizes varied among loci, particularly for the favorable homozygous CC genotype at S1_24197219 (n = 2), which may have reduced the statistical power to detect differences among genotype classes. In the AYT population, only the TT and CT genotypes were observed for marker S12_5524524, indicating limited segregation at this locus. In the SN population, only the TT genotype was observed for marker S1_24197219 (**Figure 8a**), indicating complete fixation at this locus and precluding comparisons among genotype classes. For markers S6_20589894 (**Figure 8b**) and S12_5524524 (**Figure 8c**), all expected genotype classes were observed in the SN population; however, no significant differences in DMC were detected among genotype classes (*P* > 0.05).

For markers associated with low hydrogen cyanide (HCN) content, the favorable alleles at loci S16_773999 and S14_6050078 were A and G, respectively (**Figure 9**). Genotypic classes carrying these favorable alleles generally exhibited lowest median HCN concentrations than those carrying the unfavorable alleles. However, no statistically significant differences in HCN were detected among genotype classes for either marker in either the AYT or SN population (P > 0.05).

#### 3.3.2 Marker effects and percentage of variance explained

The effects of the nine trait-associated SNP markers on total carotenoid content (TCC), dry matter content (DMC), and hydrogen cyanide (HCN) were evaluated using analysis of variance (ANOVA), and their individual contributions to phenotypic variation were quantified as percentage of variance explained (PVE) in the Advanced Yield Trial (AYT) and Seedling Nursery (SN) populations (**Supplementary Tables 2**, **3**).

For TCC, marker S1_24155522 showed a highly significant effect (p < 0.001) in the AYT population, explaining 29.94% of the phenotypic variance. The remaining TCC markers (S1_30543962, S5_3387558, and S8_25598183) exhibited low PVE values ranging from 1.71% to 6.19% and were not statistically significant. In the SN population, all TCC markers explained only a small proportion of the phenotypic variance showed very low PVE values (<1%), with no significant effects detected.

For DMC, markers S1_24197219, S6_20589894, and S12_5524524 explained only a small proportion of the phenotypic variance in the AYT population (PVE = 0.02-9.22%), and none of the marker effects were statistically significant. Similarly, in the SN population, all three markers explained less than 0.3% of the phenotypic variance, with no significant marker effects detected.

For HCN, marker S16_773999 explained 24.34% of the phenotypic variance in the AYT population, although the marker effect was not statistically significant (p > 0.05). Marker S14_6050078 explained 2.25% of the phenotypic variance in the same population. In the SN population, both markers exhibited very low PVE values (≤ 1.08%), and neither marker showed a statistically significant effect. Across all traits, the SN population consistently exhibited lower PVE values than the AYT population. With the exception of S1_24155522 for TCC, none of the evaluated markers showed statistically significant associations with the studied traits.

#### 3.3.3 Prediction accuracy of marker-based models

**Figure 10:**
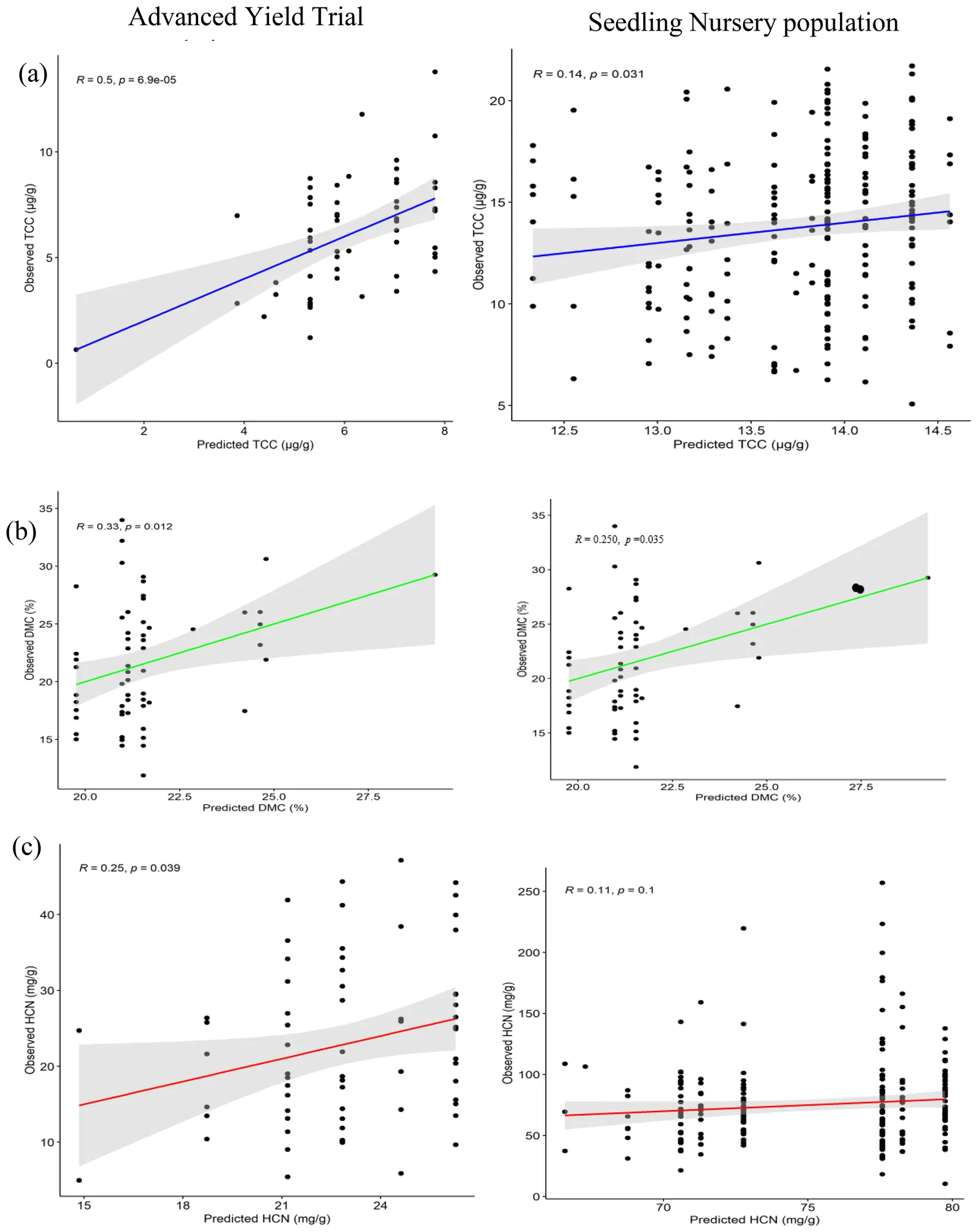
Scatter plots showing the correlation between observed and marker-predicted values for total carotenoid content (TCC; iCheck fluoro) **(a)**, dry matter content **(b)**, and hydrogen cyanide **(c)** in the Advanced Yield Trial (AYT) and Seedling Nursery (SN) populations. Pearson’s correlation coefficients (r) and corresponding significance levels are shown for each population.

The predictive performance of SNP marker-based models for total carotenoid content (TCC), dry matter content (DMC), and hydrogen cyanide (HCN) was evaluated by correlating marker-predicted values with observed phenotypic measurements (**Figure 10**). For TCC, a moderate and significant positive correlation was observed in the Advanced Yield Trial (AYT) population (*r* = 0.50, *p* < 0.05), indicating reasonable predictive accuracy of the marker-based model (**Figure 10a**). In contrast, the Seedling Nursery (SN) population exhibited a weak but significant correlation (*r* = 0.14, *p* < 0.05), suggesting reduced predictive performance at early breeding stages (**Figure 10a**).

For DMC, marker-based predictions showed a significant positive correlation with observed values in both populations, with moderate predictive ability in the AYT (r = 0.33, *p* < 0.05) and relatively lower but still significant correlation in the SN population (r = 0.25, *p* < 0.05).

For HCN, a weak but significant positive correlation was observed in the AYT population (r = 0.25, *p* < 0.05). However, in the SN population, the relationship between predicted and observed values was weak and not statistically significant (r = 0.11, *p* > 0.05), indicating poor predictive performance for HCN at this stage. Overall, correlation coefficients were consistently higher in the AYT population than in the SN population across the three evaluated traits.

## 4 Discussion

This study evaluated phenotypic variation and validated SNP markers associated with total carotenoid content (TCC), dry matter content (DMC), and hydrogen cyanide (HCN) across two independent cassava breeding populations. The substantial phenotypic variation observed for these traits indicates the presence of exploitable genetic diversity for simultaneous improvement of nutritional quality, processing attributes, and food safety through molecular breeding. The continuous distribution of TCC, DMC, and HCN further supports their quantitative inheritance, which reflects the combined influence of multiple genes and environmental factors. Similar patterns of phenotypic variation have been reported in previous cassava breeding populations (Carvalho et al., 2016; Ceballos et al., 2017; Anokye et al., 2026). Furthermore, positive skewness observed for TCC in the Advanced Yield Trial suggests that relatively few genotypes possessed exceptionally high carotenoid concentrations, a pattern commonly observed in breeding populations undergoing directional selection for provitamin A biofortification. Moderate DMC observed across most genotypes may partly reflect environmental conditions during crop development, particularly rainfall before harvest, which has previously been associated with reductions in cassava dry matter content (Belalcazar et al., 2016).

The contrasting relationships observed between TCC and DMC across the two populations further highlight the influence of genetic background and environmental conditions on trait expression. Whereas a weak negative association was observed in the AYT population, a moderate positive correlation was detected in the SN population. These contrasting patterns suggest that the relationship between TCC accumulation and DMC is population-dependent rather than universally antagonistic, which support previous reports that simultaneous improvement of both traits is achievable through appropriate parental selection and breeding strategies (Ceballos et al., 2013; Esuma et al., 2016). The variation in HCN content observed across the two populations reflects the combined influence of genetic and environmental factors on cyanogenic potential in cassava. Although clear differences in HCN concentration were observed between the Advanced Yield Trial (AYT) and seedling nursey (SN) populations, the AY population generally exhibited lower cyanide concentrations than the SN population. Most AY genotypes and many SN genotypes fell within the range commonly reported for improved cassava germplasm and “sweet” cassava cultivars (≤100 ppm), although a subset of SN genotypes exceeded this threshold (Sánchez et al., 2009; Ospina et al., 2021; FAO/WHO, 2005; Ceballos et al., 2017). Likewise, while all AYT genotypes remained below the recommended maximum level of approximately 15 mg 100 g^−1^ (150 ppm) for fresh cassava roots intended for table consumption, several SN genotypes exceeded this level. These findings indicate that cyanogenic potential varied substantially among the evaluated germplasm, emphasizing the importance of continued selection for low-HCN genotypes in cassava breeding programs and appropriate processing before consumption (Codex Alimentarius Commission, 2019; Ceballos et al., 2017). The wider distribution of HCN concentrations in the SN population is expected because it represents an early-generation breeding population with greater phenotypic segregation than the more advanced AYT population.

The differences in HCN concentration between the AYT and SN populations are likely attributable to variation in harvest age (12 vs. 8 month after planting), environmental conditions, geographical location, nitrogen availability, seasonal effects, and water stress, all of which are known to influence cyanogenic glucoside accumulation in cassava (Chávez et al., 2005; Padonou et al., 2005; Abraham et al., 2016; Peprah et al., 2020; FAO, 2020). These findings indicate that although HCN is under genetic control, its phenotypic expression is strongly influenced by environmental conditions, emphasizing the importance of evaluating cyanide-associated markers across multiple environments before their routine deployment in marker-assisted selection. The relatively low heritability observed for DMC and the population-specific differences in HCN further support the need for multi-environment validation to identify markers that remain stable across diverse genetic backgrounds and production environments.

The high within-trial broad-sense heritability estimates (H^2^) observed for TCC and HCN indicate strong genetic factors accounted for a substantial portion of the phenotypic variation in these traits, suggesting that they can be effectively improved through both phenotypic and marker-assisted selection. High heritability generally increases selection efficiency because phenotypic performance more closely reflects the underlying genetic potential of individual genotypes. In contrast, the within-trial broad-sense heritability estimated for DMC reflects a stronger environmental influence and genotype-by-environment interactions, consistent with the complex quantitative inheritance of this trait. Similar patterns have been reported in previous cassava studies, where TCC is largely controlled by major-effect loci, whereas DMC is regulated by multiple small-effect loci whose expression is strongly influenced by environmental conditions (Udoh et al., 2017; Rabbi et al., 2017; Ogbonna et al., 2020a; Ige et al., 2021).

Consequently, although MAS can accelerate genetic improvement for traits with relatively simple genetic architecture, such as TCC, improvement of DMC is likely to require complementary breeding strategies, including genomic selection, multi-environment evaluation, and repeated phenotypic assessment to accurately capture its genetic potential. Therefore, traits with high heritability, such as TCC and HCN, are more amenable to early-generation selection, whereas reliable improvement of DMC requires evaluation across diverse environments and breeding cycles to account for environmental variation.

The relationships among studied traits provide Important insights into potential for simultaneous genetic improvement through indirect selection (**Figure 4**). Although the magnitude and direction of trait correlations differed between the two populations, the observed relationships collectively highlight the influence of genetic background and environmental conditions on trait expression. The positive associations observed among most traits are generally consistent with previous reports in cassava. For example, Chávez et al. (2005) reported a negative correlation (r = −0.222**) between HCN and DMC and a positive correlation (r = 0.305**) between HCN and TCC. However, this contrasts with Ceballos et al. (2017), who reported a weak negative relationship between TCC and HCN (r² = 0.15)., illustrating that correlations among these traits may vary across breeding populations.

The contrasting correlations between TCC and DMC observed in the AYT and SN populations further demonstrate that these associations are population-dependent rather than universal. In the population, a weak negative correlation between TCC and DMC was observed, which agrees with previous studies reporting an inverse association between carotenoid accumulation and dry matter content (Esuma et al., 2016; Udoh et al., 2017; Rabbi et al., 2020). Conversely, the moderate positive correlation observed in the SN population supports the findings of Ortiz et al. (2011) and Esuma et al. (2016), indicating that simultaneous improvement of TCC and DMC is achievable in appropriately selected genetic backgrounds. Differences in parental composition, selection history, allele frequencies, and environmental conditions between the AYT and SN populations likely contributed to these contrasting correlation patterns. Together, these findings suggest that unfavorable relationships between TCC and DMC are not inevitable and that breeding populations should be evaluated individually when designing multi-trait selection strategies. provide important insights into the feasibility of simultaneous genetic improvement of multiple traits through indirect selection (**Figure 4**). Although the magnitude and direction of correlations differed between populations, the observed relationships generally reflect the combined influence of genetic background and environmental conditions on trait expression.

In the AYT population, positive associations were observed between TCC (iCheck) and HCN, as well as between DMC and HCN, which may reflect the prevalence of yellow-rooted genotypes in regions such as the Amazon basin and coastal areas where highly cyanogenic lines are traditionally preferred (Chávez et al., 2005; Ogbonna et al., 2021). Conversely, the negative relationship between TCC (iCheck and visual score) and DMC indicates that genotypes with higher starch accumulation tend to exhibit reduced carotenoid concentration, likely due to dilution effects (Esuma et al., 2016; Udoh et al., 2017; Rabbi et al., 2020). However, contrasting evidence from Ortiz et al. (2011), who reported a strong positive correlation (r = 0.62) in Latin American germplasm, suggests that these relationships are context dependent.

Such variation in trait associations may be explained by differences in allelic composition at loci controlling carotenoid biosynthesis and dry matter accumulation. Udoh et al. (2017) reported co-localization of QTLs for total carotenoid and dry matter content on chromosome 1, suggesting possible pleiotropic effects or tight genetic linkage. This complex genetic interplay underscores the need for comprehensive evaluation of trait relationships to inform breeding strategies aimed at simultaneously improving nutritional quality and agronomic performance.

The technical validation of KASP assays demonstrated high call rates and clear genotype clustering, confirming the reliability of the markers for genotyping applications. However, biological validation revealed that marker performance varied across traits and populations. These population-specific differences likely reflect variation in allele frequencies, genetic background, and segregation patterns between the AYT and SN populations, highlighting the importance of validating candidate markers across independent breeding populations before routine deployment in marker-assisted selection. Among TCC-associated markers, S1_24155522 showed a strong and significant effect in the AYT population, explaining a substantial proportion of phenotypic variance. This marker is linked to phytoene synthase (PSY), a key enzyme in carotenoid biosynthesis, supporting its biological relevance (Welsch et al., 2010; Rabbi et al., 2020). The markers employed across the trials demonstrated high efficacy in distinguishing genotypes carrying specific homozygous and heterozygote alleles for the three traits under study. This discrimination capability highlights the utility of SNP genotyping in detecting significant polymorphisms, which facilitates the identification of genotypic classes in early breeding selection stages (Graves et al., 2015). The allele frequencies revealed significant variations in allele substitution effects across all nine trait-linked markers for the studied traits, reflecting diverse genetic compositions within the populations examined.

Notably, markers associated with TCC demonstrated dominant gene effects, with genotypes harboring favorable alleles co-segregating with high carotenoid content (de Carvalho et al., 2022). Conversely, markers linked with DMC exhibited more complex segregation patterns, with some markers predicting heterozygotes and others identifying genotypes with favorable alleles. Additionally, markers associated with HCN levels showed fewer genotypes carrying favorable alleles, particularly marker S14_6050078, indicating potential challenges in breeding for reduced cyanogenic potential. Nevertheless, individuals with two copies of the favorable allele consistently exhibited low HCN levels, validating the efficacy of these markers in predicting phenotypic outcomes.

Similarly, the non-significant differences among genotypes for SNPs S1_30543962, S5_3387558, and S8_25598183 may reflect the limited segregation of these loci, resulting from shared alleles among the parental lines and the relatively narrow genetic base of the Seedling Nursery population. However, SNP S1_24155522 revealed a strong and significant association with TCC in the AYT population, suggesting its potential use as a marker for selecting high-TCC genotypes.

The observed differences in TCC among genotypic classes further support the association between S1_24155522 and carotenoid accumulation in cassava roots, consistent with previous studies (Esuma et al., 2016). Specifically, the locus S1_24155522 (A/C), linked to Manes 01G124200 (Phytoene synthase), a key enzyme in the carotenoid biosynthetic pathway, particularly β-carotene, plays a critical role in carotenoid biosynthesis due to its transferase enzymatic activity (Welsch et al., 2010; Esuma et al., 2016; Rabbi et al., 2017, 2020). Moreover, the proximity of locus S1_24159585, associated with color intensity, to phytoene synthase 2 (PSY2) in the cassava v. 6 reference genome suggests its involvement in carotenoid biosynthesis (Welsch et al., 2010; Rabbi et al., 2014b; Bevan et al., 2017).

The limited segregation observed for loci within the SN population underscores the complexity of dry matter content, which is often influenced by multiple loci and environmental factors. Udoh et al. (2017) reported the association of specific alleles at position 572 in the PSY2 gene with yellow- and white-root color genotypes, emphasizing the pivotal role of PSY2 in carotenoid accumulation and root coloration in cassava.

The allelic substitution effects observed for DMC indicate that locus S1_24197219 is associated with variation in dry matter content, as evidenced by the significant differences among genotypic classes in the AYT population. This indicates that the marker is associated with locus having a measurable effect on DMC expression. However, the lack of segregation observed for this marker in the SN population indicates possible allele fixation, likely resulting from prior selection within the breeding pipeline (Ige et al., 2022). Such fixation reduces genetic variability and limits the ability to detect marker effects at early breeding stages. The limited segregation also resulted in relatively small genotype classes for some alleles, which should be considered when interpreting marker effects. These contrasting patterns between populations highlight the importance of evaluating marker performance across diverse genetic backgrounds. Furthermore, the observed variability suggests that DMC is influenced by multiple loci and environmental factors, necessitating further investigation into the underlying genetic architecture. These findings improve our understanding of the genetic architecture of DMC and provide useful information for designing breeding strategies aimed at simultaneously improving DMC and total carotenoid content (TCC) in cassava.

The observed marker effects on hydrogen cyanide (HCN) content indicate that favorable and unfavorable alleles occurred in both homozygous and heterozygous genotype classes, whereas the reference allele was more frequently observed among genotypes with lower cyanogenic potential (Gleadow and Møller, 2014; Ogbonna et al., 2020a). The moderate HCN levels observed in heterozygous genotypes in the AYT population are consistent with findings by Ogbonna et al. (2020a), who reported a missense mutation at locus S16_773999 (A to G substitution in exon 4) leading to an amino acid change from threonine to alanine. The generally low cyanogenic potential observed across AYT genotypes likely reflects the genetic background of West African germplasm and the effects of domestication and recurrent selection, which have reduced cyanogenic glucoside content (Ramu et al., 2017; Ogbonna et al., 2020b). In contrast, the relatively higher HCN levels observed in some SN families may be attributed to the earlier harvest stage (8 months after planting), as cyanogenic content is known to vary with plant age and environmental conditions (Nzwalo and Cliff, 2011; Hue et al., 2012). These findings further support the need to validate HCN-associated markers across multiple environments and breeding populations before routine implementation in marker-assisted selection. Accordingly, additional validation across multiple environments and breeding cycles will be valuable for confirming the stability of these marker effects before routine application in marker-assisted selection.

In contrast, most markers associated with DMC and HCN exhibited low PVE and were not statistically significant, indicating limited predictive ability. This is consistent with the polygenic nature of these traits and the influence of genotype-by-environment interactions (Ceballos et al., 2015; Esuma et al., 2022). The decline in marker performance from the AYT to the SN population underscores the need to validate candidate markers across diverse genetic backgrounds and breeding stages (Collard and Mackill, 2008; Andelkovic et al., 2020; Kanaabi et al., 2024). The relatively high PVE and association observed for marker S1_24155522 support its potential utility as a candidate marker for improving carotenoid content in cassava. However, the weak performance of most markers suggests that individual markers explained only a small proportion of the observed phenotypic variation and should be used cautiously. Integrating multiple validated markers or complementary genomic selection approaches may provide more reliable selection for these complex traits.

Prediction accuracy analysis revealed moderate correlations between marker-predicted and observed values for TCC and DMC, but comparatively weak performance for HCN, particularly in the SN population (Resende et al., 2010; Esuma et al., 2016; Ige et al., 2022). These findings indicate that validated SNP markers can provide useful information for early-stage selection, particularly for carotenoid content, although their predictive ability remains limited for complex traits influenced by multiple loci and environmental factors. Consequently, combining validated candidate markers with genome-wide prediction approaches may further improve prediction accuracy and enhance breeding efficiency.

This study focused on validating previously reported candidate KASP markers rather than identifying novel marker–trait associations. Although the use of two independent breeding populations strengthened the biological validation of these markers, some genotype classes contained relatively few individuals because of low allele frequencies or marker fixation, reducing statistical power for selected comparisons. Furthermore, marker performance and within-trial broad-sense heritability estimates were obtained from a single growing season, and additional multi-environment validation will be valuable before routine deployment of these markers in marker-assisted selection.

## Data Availability

The datasets generated and analyzed during this study, including the phenotypic and genotypic data supporting the findings of this work, have been deposited in the Zenodo repository and are publicly available at https://doi.org/10.5281/zenodo.21469711. Additional supporting information is provided in the article and its **Supplementary Material.**

## Supplementary material

Supplementary Table 6 Markers and flanking sequences of the SNPs used for genotyping of cassava genotypes in the two populations._*Chr. = chromosome position, * The positions of SNP markers, expressed in base pairs (bp), were determined using the cassava reference genome version 6.1 (Bredeson et al., 2016; Anokye et al., 2026).
