# Supplementary material for: Validation of single nucleotide polymorphism markers associated with hydrogen cyanide, total carotenoid and dry matter contents in cassava (Manihot esculenta Crantz)
Source: Front Plant Sci. 2026 Aug 7;17:1912608. doi: 10.3389/fpls.2026.1912608 (PMC13493511; doi:10.3389/fpls.2026.1912608)
Supplement: Supplementary file 1 [file DataSheet1.docx]

**Supplementary**

## Advanced yield trial, Seedling nursery trial Progenitors

**S1_24155522**

**S1_30543962**

**S1_**

**3387558**

**SS8_**

**25598183**

Supplementary Figure 1: Allelic discrimination plots of KASP assays for SNP markers linked to carotenoid content across the two breeding populations and the parents.

*NTC: non-template control

## Advanced yield trial Seedling nursery trial Progenitors

**SS1_24197219**

**SS6_20589894**

**SS12_5524524**

Supplementary Figure 2: Allelic discrimination plots of KASP assays for SNP markers linked to dry matter content (DMC) across the advanced yield trial, seedling nursery population, and progenitors. *NTC: non-template control

## Advanced yield trial Seedling nursery trial Progenitors

**SS16_773999**

**SS14_6050078**

Supplementary Figure 3: Allelic discrimination plots of KASP assays for SNP markers linked to hydrogen cyanide content across the two breeding populations and the progenitors.

*NTC: non-template control

**Supplementary Table 1**. Number of predicted genotypes for markers linked to carotene content, dry matter content, and hydrogen cyanide content in the advanced yield trial and seedling nursery population.

| S1_24155522 (TCC) | | | | |
| --- | --- | --- | --- | --- |
| AA | CA | CC | Missing values | Trial |
| 52 (68.4) | 16 (21.1) | 3 (4.0) | 5 (6.5) | AYT |
| 259 (100.0) |  |  |  | SN |
| S1_30543962 (TCC) | | | | |
| GG | GA | AA |  |  |
| 8 (10.5) | 47 (61.8) | 20 (26.4) | 1 (1.3) | AYT |
| 29 (11.2) | 135 (52.3) | 94 (36.4) | 1 (0.3) | SN |
| S5_3387558 (TCC) | | | | |
| TT | TC | CC |  |  |
| 1 (1.3) | 66 (86.8) | 9 (11.8) |  | AYT |
|  | 188 (72.6) | 71 (27.4) |  | SN |
| S8_25598183 (TCC) | | | | |
| TT | TG | GG |  |  |
| 4 (5.5) | 27 (36.9) | 42 (57.5) | 3 (3.9) | AYT |
| 38 (14.7) | 148 (57.1) | 72 (27.8) | 1 (0.4) | SN |
| S1_24197219 (DMC) | | | | |
| CC | TC | TT |  |  |
| 2 (2.6) | 13 (17.1) | 60 (79.0) | 1 (1.3) | AYT |
|  |  | 259 (100.0) |  | SN |
| S6_20589894 (DMC) | | | | |
| GG | GA | AA |  |  |
| 25 (32.9) | 27 (35.5) | 14 (18.4) | 10 (13.2) | AYT |
| 20 (7.7) | 137 (52.9) | 101 (39.0) | 1 (0.4) | SN |
| S12_5524524 (DMC) | | | | |
| TT | CT | CC |  |  |
| 49 (64.5) | 21 (27.6) |  | 6 (7.9) | AYT |
| 91 (35.1) | 151 (58.3) | 17 (6.6) |  | SN |
| S16_773999 (HCN) | | | | |
| AA | GA | GG |  |  |
| 8 (10.5) | 27 (35.6) | 38 (50.0) | 3 (3.9) | AYT |
| 96 (37.1) | 108 (41.7) | 54 (20.8) | 1 (0.4) | SN |
| S14_6050078 (HCN) | | | | |
| GG | AG | AA |  |  |
| 2 (2.7) | 25 (32.9) | 46 (60.5) | 3 (3.9) | AYT |
| 13 (5.0) | 104 (40.2) | 141 (54.4) | 1 (0.4) | SN |

***DMC = dry matter content; TCC = total carotene content; HCN = hydrogen cyanide; AYT = Advanced yield trial; SN = seedling nursery trial.

**Supplementary Table 2:** Residual and total sum of squares (SS) for total carotenoid content (TCC), dry matter content (DMC), and hydrogen cyanide (HCN) across advanced yield trial (AYT) and seedling nursery (SN) populations in cassava.

| Population | Residual SS | Total SS |
| --- | --- | --- |
| **TCC (AYT)** | 279.288 | 439.420 |
| **TCC (SN)** | 3217.500 | 3283.410 |
| **DMC (AYT)** | 1368.831 | 1531.876 |
| **DMC (SN)** | 12488.420 | 12517.120 |
| **HCN (AYT)** | 7249.545 | 9748.983 |
| **HCN (SN)** | 272067.840 | 275255.020 |

SS = sum of squares; AYT = advanced yield trial; SN = seedling nursery population; TCC = total carotenoid content; DMC = dry matter content; HCN = hydrogen cyanide content. Residual SS represents unexplained variation, while total SS denotes the overall phenotypic variance within each population.

**Supplementary Table 3**: Mean squares (MS), sum of squares (SS), and percentage of variance explained (PVE) of SNP markers associated with total carotenoid content (TCC), dry matter content (DMC), and hydrogen cyanide (HCN) evaluated in cassava genotypes across advanced yield trial (AYT) and seedling nursery populations.

|  |  | Advanced yield trial | |  |  |  | Seedling nursery population | |  |
| --- | --- | --- | --- | --- | --- | --- | --- | --- | --- |
| Trait | Marker | df (AYT) | MS (AYT) | SS (AYT) | PVE (%) (AYT) | df (SN) | MS (SN) | SS (SN) | PVE (%) (SN) |
| **TCC** | S1_24155522 | 2 | 59.698*** | 119.396 | **29.94** | NA | NA | NA | NA |
|  | S1_30543962 | 2 | 2.426ⁿˢ | 4.852 | 1.71 | 2 | 13.060ⁿˢ | 26.120 | 0.81 |
|  | S5_3387558 | 2 | 9.210ⁿˢ | 18.420 | 6.19 | 1 | 25.510ⁿˢ | 25.510 | 0.79 |
|  | S8_25598183 | 2 | 8.732ⁿˢ | 17.464 | 5.88 | 2 | 7.140ⁿˢ | 14.280 | 0.44 |
| **DMC** | S1_24197219 | 2 | 69.504ⁿˢ | 139.008 | 9.22 | NA | NA | NA | NA |
|  | S6_20589894 | 2 | 11.915ⁿˢ | 23.830 | 1.71 | 2 | 13.670ⁿˢ | 27.340 | 0.22 |
|  | S12_5524524 | 1 | 0.207ⁿˢ | 0.207 | 0.02 | 2 | 0.680ⁿˢ | 1.360 | 0.01 |
| **HCN** | S14_6050078 | 2 | 83.355ⁿˢ | 166.710 | 2.25 | 2 | 1484.140ⁿˢ | 2968.280 | 1.08 |
|  | S16_773999 | 2 | 1166.364ⁿˢ | 2332.728 | **24.34** | 2 | 109.450ⁿˢ | 218.900 | 0.08 |

*** = p < 0.001; ** p < 0.01; * = p < 0.05; ^sn^ = not significant; df = degrees of freedom; MS = Mean Squares; PVE = percentage variation explained; AYT = Advanced yield trial; SN = Seedling nursery; TCC = total carotene content; DMC = dry matter content; HCN = hydrogen cyanide content.

**Supplementary Table 4**: Within-family performance and selection index in the SN population for dry matter (DMC), total carotenoid content (TCC), and hydrogen cyanide potential (HCN), including their pedigree.

| Pedigree | Families | Average_DMC | Average_TCC | Average_HCN | SI |
| --- | --- | --- | --- | --- | --- |
| IITA-TMSIBA180231/ IITATMS-IBA150815 | IBASN201112 | 32.45 | 15.81 | 69.52 | 6.14 |
| IITA-TMSIBA180065/ IITATMS-IBA180088 | IBASN201136 | 27.75 | 16.49 | 82.27 | 5.93 |
| IITA-TMSIBA170067/ IITATMS-IBA180106 | IBASN201156 | 30.45 | 14.77 | 92.23 | 5.76 |
| IITA-TMSIBA154810/ IITATMS-IBA180158 | IBASN201098 | 31.08 | 14.58 | 78.14 | 5.73 |
| IITA-TMSIBA141092/ IITATMS-IBA180106 | IBASN201149 | 29.20 | 14.35 | 75.91 | 5.53 |
| IITA-TMSIBA180158/ IITATMS-IBA180106 | IBASN201160 | 29.04 | 14.05 | 84.73 | 5.47 |
| IITA-TMSIBA180047/ IITATMS-IBA180081 | IBASN201129 | 26.94 | 14.79 | 56.48 | 5.42 |
| IITA-TMSIBA180148/ IITATMS-IBA180081 | IBASN201125 | 27.54 | 14.51 | 62.08 | 5.41 |
| IITA-TMSIBA180146/ IITATMS-IBA180071 | IBASN201144 | 30.16 | 13.64 | 45.75 | 5.37 |
| IITA-TMSIBA164800/ IITATMS-IBA182939 | IBASN201106 | 28.21 | 14.14 | 99.97 | 5.37 |
| IITA-TMSIBA180017/ IITATMS-IBA180256 | IBASN201143 | 29.06 | 13.56 | 78.79 | 5.34 |
| IITA-TMSIBA154798/ IITATMS-IBA150815 | IBASN201092 | 36.20 | 11.46 | 37.43 | 5.29 |
| IITA-TMSIBA180148/ IITATMS-IBA180106 | IBASN201159 | 27.79 | 13.44 | 84.20 | 5.22 |
| IITA-TMSIBA180180/ IITATMS-IBA180106 | IBASN201076 | 26.04 | 14.26 | 81.66 | 5.21 |
| IITA-TMSIKN160021/ IITA-TMS-IBA150815 | IBASN201121 | 25.68 | 13.55 | 84.68 | 5.09 |
| IITA-TMSIBA150605/ IITATMS-IBA150815 | IBASN201089 | 31.96 | 11.12 | 66.25 | 4.99 |
| IITA-TMSIBA180065/ IITATMS-IBA180106 | IBASN201158 | 27.27 | 11.48 | 68.62 | 4.69 |
| IITA-TMSIKN130010/ IITA-TMS-IBA150815 | IBASN201118 | 30.29 | 9.77 | 57.79 | 4.52 |

| Genotype | DMC (%) | HCN (mg 100 g⁻¹) | TCC (μg/g) | SI |
| --- | --- | --- | --- | --- |
| UIC-17-265 | 23.70 | 41.92 | 13.79 | 7.67 |
| UIC-17-122 | 34.00 | 13.12 | 7.65 | 6.40 |
| UIC-17-464 | 21.38 | 16.21 | 10.76 | 6.11 |
| UIC-17-325 | 17.45 | 21.64 | 11.78 | 6.09 |
| UIC-17-2402 | 28.68 | 24.72 | 7.84 | 6.02 |
| UIC-17-257 | 26.03 | 18.06 | 8.30 | 5.80 |
| UIC-17-174 | 24.67 | 13.49 | 8.85 | 5.74 |
| UIC-17-2831 | 25.56 | 10.27 | 8.71 | 5.68 |
| TMS01/1368 | 28.26 | 20.40 | 7.05 | 5.64 |
| UIC-17-1677 | 24.97 | 63.25 | 6.98 | 5.39 |
| TMS07/0593 | 20.14 | 39.94 | 8.57 | 5.35 |
| TMS97/0593 | 24.23 | 37.99 | 7.21 | 5.33 |
| UIC-17-2823 | 17.89 | 19.02 | 9.62 | 5.33 |
| UIC-17-1632 | 17.16 | 17.25 | 9.21 | 5.07 |
| UIC-17-2778 | 17.91 | 34.35 | 8.33 | 4.98 |
| UIC-17-1119 | 20.94 | 14.15 | 7.85 | 4.96 |
| UIC-17-750 | 29.66 | 25.78 | 4.53 | 4.94 |
| UIC-17-1343 | 27.19 | 11.86 | 5.94 | 4.93 |
| UIC-17-1048 | 20.84 | 26.61 | 7.36 | 4.93 |
| UIC-17-2074 | 22.43 | 29.46 | 6.76 | 4.92 |
| UIC-17-2089 | 16.88 | 26.39 | 8.43 | 4.85 |
| UIC-17-25 | 20.84 | 18.68 | 7.32 | 4.84 |
| UIC-17-8 | 30.64 | 29.55 | 3.83 | 4.82 |
| UIC-17-220 | 15.92 | 19.32 | 8.75 | 4.79 |
| UIC-17-662 | 18.24 | 42.55 | 7.60 | 4.78 |
| UIC-17-119 | 18.97 | 21.00 | 7.53 | 4.72 |
| UIC-17-679 | 23.60 | 22.82 | 5.77 | 4.65 |
| UIC-17-1713 | 30.30 | 30.57 | 3.40 | 4.64 |
| UIC-17-2432 | 29.09 | 11.39 | 4.13 | 4.48 |
| UIC-17-93 | 22.89 | 38.44 | 5.19 | 4.45 |
| UIC-17-2259 | 23.26 | 25.93 | 5.09 | 4.39 |
| UIC-17-707 | 21.92 | 28.10 | 5.29 | 4.32 |
| UIC-17-986 | 18.44 | 26.25 | 6.31 | 4.27 |
| UIC-17-1200 | 19.81 | 9.05 | 6.73 | 4.22 |
| UIC-17-2511 | 15.19 | 18.52 | 7.39 | 4.20 |
| UIC-17-746 | 15.46 | 25.18 | 6.98 | 4.15 |
| UIC-17-1453 | 17.39 | 26.50 | 6.29 | 4.14 |

**Supplementary Table 5**: BLUE values and selection index for biofortified yellow roots clones in the AYT for total carotene content (TCC), dry matter content (DMC) and low hydrogen cyanide potential (HCN)

Supplementary Table 6: Markers and flanking sequences of the SNPs used for genotyping of cassava genotypes in the two populations

| Marker_allele_trait | **Chr.** | **SNPs and Sequence** |
| --- | --- | --- |
| S1_24197219 (C/T) | 1 | GATGTAGGCATGTTACATATAAGGGCTACATACACATTAGCAGCTAAAATGAGACCCGGATACCGAGCAATGCCATCAATTGAGAGATGAACTCAGGGTG[C/T]CCTGGCCATGCAGCTCCAGTAACCAAATTTTCATGAGTGTAGCAACGATGTATTGGATCAGGTTCTAGCCATGTTGCCCCAGCCAAGACCACGTTAATCT |
| S6_20589894 (A/T) | 6 | ATTGATGATTTTTTATTCATGATATGTAGCTATCAAAGTTACTCAGCAATGTCCTTGTTTTAGCCATGCTAGCAGCATGTTTTGTTGCGACAACAGTTGG[A/G]AGTTGTATGAATATTGTTTTATCTTGTATGCAGAATATCATTGGGCAGGAAGCAGGGAAAAGCGTGATTGAGGAATATTTACGTCGTAGGGGTCACTCAG |
| S1_30543962 (A/G) | 1 | GGAGGTTTTTTTATGTGGCATTCTCAGCAGCTGCAGGAATCTCATTGTTCTTTACAATTCCAAGGCTCTTTCTTGCAATTAAAGGTGGGGAAGGTGCCCC[A/G]GACCTCTGGGGAACTGCTGGAAATGCTGCCATTAATATTGGTGGTAAATGCTTTAACCTTTCTCTGTCATATGAAGAAAATGAGTTAATTGATGTATAAT |
| S5_3387558 (C/T) | 5 | GTTACACTTAGACCCTTGTCATTAAACATTACTGAGGCTGCAGTTGAAGTGTAAACAACTCTTTTCACTGTCTTTGATTCCAAGCATGTCCTTAATATCC[C/T]TAGCAATCCATCCACGGCTATTTTGGTCACACTTTCTTCAGGTTCTTTTCCATAATGATCCATTGGGTGAGCCACATGGAAGACTCCAATACAACCTTCA |
| S8_25598183 (G/T) | 8 | TAAATTCTGACTGTCTTGGCATGACTGTCCAGGTAGTCCCCGAAAATGAGAATGCTGCTCTCTACTCCACTCATTCATTCAAGATTTTGTTCAAGGAAGG[G/T]GGTTGTGGAACCTTCATTCCGCTCTTTTTCAACTTGCTCTCTTCAGTAAGGCAATACAATCAGCAACAAACCTCTGGAATGGGGCCCCAGATGAACCCTT |
| S1_24155522 [A/C] | 1 | GACAGATGAGCTTGTTGATGGACCTAATGCTTCACACATAACGCCAACAG[A/C]TTTAGATAGGTGGGAAGCAAGGTTGGAAGATATGTTTCGAGGTCGTCCCT |
| S12_5524524 [T/C] | 12 | TGAATTATTTTAACTCTTTGATTGCTTCGCCAGTGCCTGGTCTCCAGAATGTGTGTGTTGCTTTGGTTTGTAGTTCCAAAGGTGAGCTGTGGCAATTTTA[T/C]TGCAGCCCCACTGGCATTAGACGCAGTAAATTATATCAGGACGAAGTAAGTTCATCCTTCAAAGGAAATGATAATGGTCAATTTGTGGGGAGCAAAGGTT |
| S16_773999 [A/G] | 16 | GTGAGCAACGAGCTAGGAGCAGGACATCCAAAATCAGCAGCATTTTCTGTGATAATTGTGAATCTATGCTCTTTCATTATCGCTGTWRTCGCAGCCATCRTCGTGATGATWTTGCGAGACTATCTCAGCTACGCTTTYACTGATGGTGAA[A/G]CTGTTTCCAAAGCRGTCTCCGATCTYACCCCCTWCTTGGCTGYCACCCTCATTCTAAATGGSGTGCAGCCTGTTTTGTCCGGTRAGATAATYYCCCCAACCTCCCCCCTGTTCCCTGTTATTATTAYGATTCCTTCATAGCAGTACTTTT |
| S14_6050078 [G/A] | 14 | CTCKGCAGGTATCTTACCCATAATTTTTTTTAWAAGGAAATACRGAGACTAAGATTTTCAAATATTTTATTCATATATGYCTAAYGTMTTAAAAATTGGCAAATTTATAGGAGCATTTGCAGCAATGGCAATATTCGGAAAGATGGACCA[A/G]TTACTTGCGCCTAAAGGAATTTCTATGACAATTGCGCCCTTAGGAGCTGTTTGTGCCGTCCTCTTTGCCACTCCTTCCTCYCCTGCTGCTCGGGTACCCTCCWCTGCTCYGTTTTATGGCAGTAGATAAAAATGAGAACTTTTTAACTTT |

_*Chr. = chromosome position, * The positions of SNP markers, expressed in base pairs (bp), were determined using the cassava reference genome version 6.1 (Bredeson *et al*., 2016; Anokye *et al*., 2026).
